# The 4-vinylcyclohexene dioxide induced mouse ovarian premature failure is caused by down regulation of IGF1R and triggering excessive autophagy

**DOI:** 10.1007/s44154-024-00197-3

**Published:** 2025-03-21

**Authors:** Yuwei Niu, Yuyang Miao, Wenjing Wan, Qiankun Wang, Yingwan Ma, Menghao Pan, Baohua Ma, Qiang Wei

**Affiliations:** 1 Key Laboratory of Animal Biotechnology, Ministry of Agriculture, Northwest A&F University, Yangling, Shaanxi 712100 China; 2 College of Veterinary Medicine, Northwest A&F University, Yangling, Shaanxi 712100 China

**Keywords:** 4-vinylcyclohexene dioxide, Autophagy, Insulin-like growth factor 1 receptor, Granulosa cells, Primordial follicles, Premature ovarian failure

## Abstract

The 4-Vinylcyclohexene dioxide (VCD) is a common occupational chemical which can lead to ovary toxicity. Autophagy is an evolutionarily conserved process that is crucial for regulating the follicular development during the whole reproductive lifespan. In order to study the effect and mechanism of VCD on autophagy in ovary, VCD was intraperitoneally injected into mouse to induce ovary toxicity model. The results showed that VCD inhibited IGF1R/AKT/mTOR signaling pathway by down-regulating the expression of IGF1R in ovarian granulosa cells (GCs) and induced autophagy in ovaries. In in vitro experiments further demonstrated that by VCD-induced IGF1R inhibition or siRNA-mediated IGF1R knockdown could trigger excessive autophagy flux in human granulosa-like tumor cell line KGN cells. Moreover, SC79, the activator of the IGF1R/AKT/mTOR signaling pathway could inhibit the excessive autophagy led by siRNA-mediated IGF1R knockdown or VCD treatment in KGN cells. Functionally, as a result of VCD-triggered excessive autophagy, VCD induced WT1 selective degradation by autophagy leading to premature differentiation of ovarian GCs and the premature activation of primordial follicles. In conclusion, VCD inhibits IGF1R/AKT/mTOR signaling pathway by downregulating the expression of IGF1R and triggers excessive autophagy in ovarian GCs, leading to abnormal GCs function and the consumption of ovarian follicle pool.

**Supplementary Information:**

The online version contains supplementary material available at 10.1007/s44154-024-00197-3.

## Introduction

The 4-Vinylcyclohexene dioxide (VCD) has been widely used in the industry (Cannady et al. 2003). Many in vivo and in vitro experiments have shown that VCD can induce ovary toxicity by triggering caspase-dependent apoptosis and accelerate the follicle atresia leading to follicle pool consumption (Hu et al. 2001; Springer et al. 1996; Takai et al. 2003). Additionally, animal model exposed to VCD undergo premature ovarian failure (POF) that mirrors the natural progression from perimenopause to menopause in humans (Hoyer & Sipes 2007; Cao et al. 2020; Gannon et al. 2023; Hubbard et al. 2023; Jiao et al. 2022; Konhilas et al. 2020; Lee et al. 2023; Tang et al. 2021).The mechanism of VCD-induced ovary toxicity is generally considered to be related to the oocytes apoptosis induced by inhibition of the AKT/mTOR signaling pathway(Chen et al. 2015; Mark-Kappeler et al. 2011; Pontifex et al. 2021). The insulin-like growth factor 1 receptor (IGF1R) is not only an important upstream regulator of the AKT/mTOR signaling pathway, but also crucial for follicle survival and development (Baumgarten et al. 2017; Sekulovski et al. 2020). Especially, the IGF1R/AKT/mTOR signaling pathway is essential for the function of ovarian granulosa cells (GCs) to support follicular development (Baumgarten et al. 2017; He et al. 2019; Zhang et al. 2022).

Recently, more and more evidences suggest that autophagy is crucial for regulating the follicular development and atresia (Bhardwaj et al. 2022; Castro-Cruz et al. 2023; Liu & Wang 2023). Whereas, insufficient or excessive autophagy may result in disruption of the ovarian function (Choi et al. 2010; Gawriluk et al. 2011; Liu et al. 2023; Song et al. 2015). It is reported that inhibition of IGF1R promotes excessive autophagy (Wu et al. 2021; Zhou et al. 2016), which can lead to autophagic cell death (ACD) or Type-II programmed cell death (Denton & Kumar 2019). Therefore, as a commonly used drug to induce POF and menopausal related diseases animal model, there is a novel hypothesis about the ovary toxicity of VCD, which is that VCD inhibits Akt/mTOR signaling pathway by down-regulating the expression of IGF1R leading to disturbance of autophagy in ovarian GCs. To verify this hypothesis, in the present study, the effects of VCD on the autophagy in mouse ovary and human granulosa-like tumor cell line KGN cells were investigated.

## Result

### The establishment of VCD induced mouse POF model

**Fig. 1:**
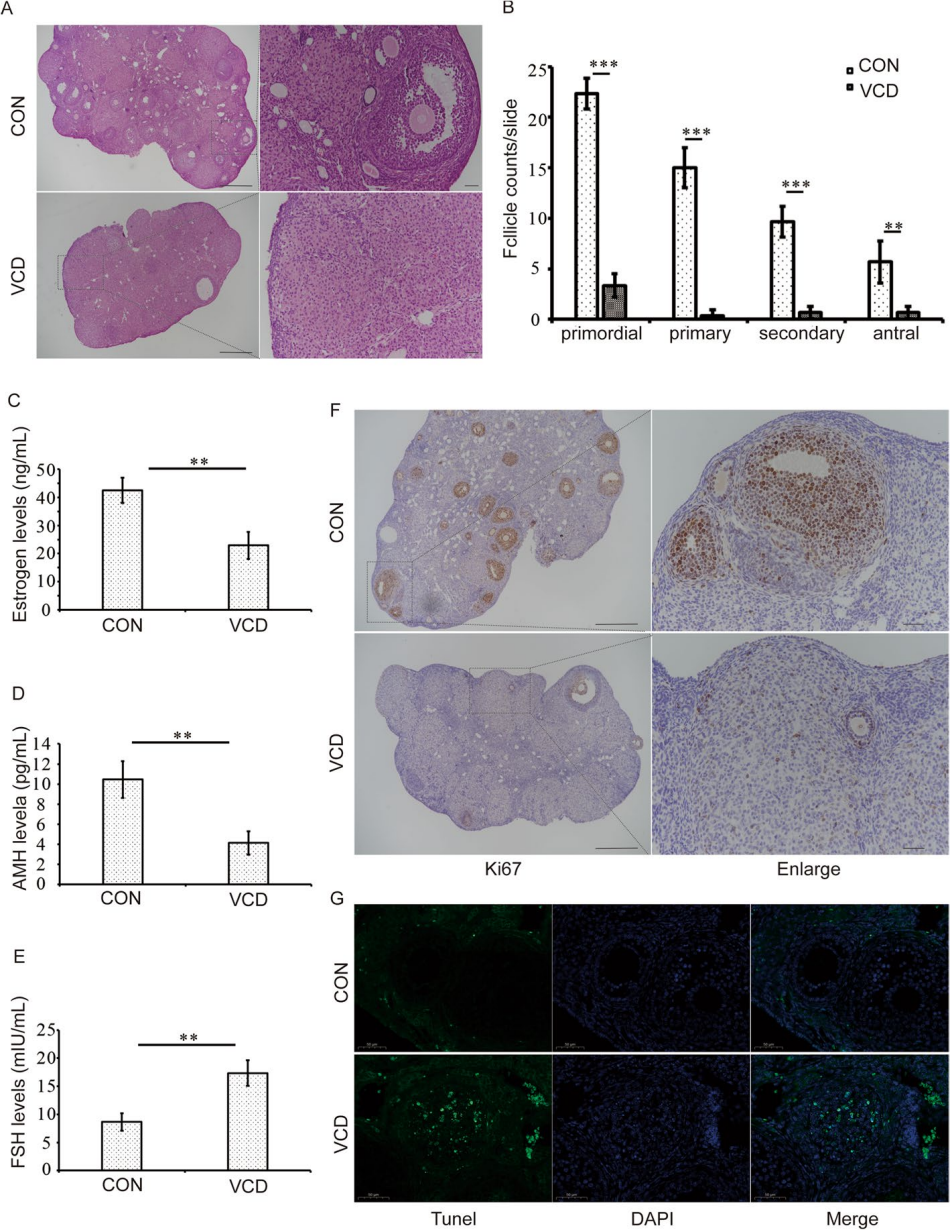
VCD induced the establishment of mouse POF model. **A** Histological observation of ovary in control and POF groups. Bar, left, 500 μm, right, 50 μm. **B** The counting results of different follicles, including primordial, primary, secondary and antral follicles in control and POF groups (*n* = 9mice/group). **C** Serum E_2_ levels in control and POF groups (*n* = 9mice/group). **D** Serum AMH level in control and POF groups(*n* = 9mice/group). **E** Serum FSH level in control and POF groups(*n* = 9mice/group). **F** Representative images of Ki67 immunohistochemistry of ovary in control and POF groups. Bar, left, 500 μm, right, 50 μm. **G** Representative images of TUNEL analysis of ovary in control and POF groups. Bar, 50 μm. E_2_ = Estrogen, AMH = anti-Mullerian hormone, FSH = Follicle-Stimulating Hormone. Each experiment was independently repeated at least three times. The results are expressed as means ± SEM. **p < 0.01, ***p < 0.001 vs control. The control group was intraperitoneally injected with the same amount of sesame oil

We used 6-week-old C57BL/6 mice to establish POF model. After 15 days of continuous VCD injection, the establishment of POF mouse model was confirmed by disordered estrous cycle detected by vaginal smears for 12 consecutive days (Fig S1 A). As shown in Fig S1 B, regular estrous cycles could be observed in control group. In the POF model group, almost all mice showed disordered estrous cycles and mainly stayed in the metaestrous phase (Fig S1 B). We then identified and counted follicles in various stages of development (Fig S2). Compared with the control group, the follicle count results revealed that the numbers of follicles in different developmental stages were significantly decreased (*p* < 0.001 for primordial, primary and secondary follicles; *p* < 0.01 for antral follicles) (Fig. 1 A, B). Meanwhile, relative hormone levels in serum were measured. Compared with the control group, the estrogen (E_2_) (*p* < 0.01) and anti-mullerian hormone (AMH) (*p* < 0.01) levels were significantly decreased (Fig. 1 C, D), and follicle-stimulating hormone (FSH) level was remarkably elevated in the POF group (*p* < 0.01) (Fig. 1 E). These changes implied the successful establishment of mouse POF model. Then, the cell proliferation and apoptosis in ovarian GCs were analyzed. In the POF group, the number of Ki67-positive cells was lower than that of the control group, while the number of TUNEL positive apoptotic cells was higher than that of the control group, indicating that VCD inhibited the proliferation and promoted apoptosis in ovarian GCs (Fig. 1 F, G).

### VCD down-regulated the expression of IGF1R in the ovarian GCs

**Fig. 2:**
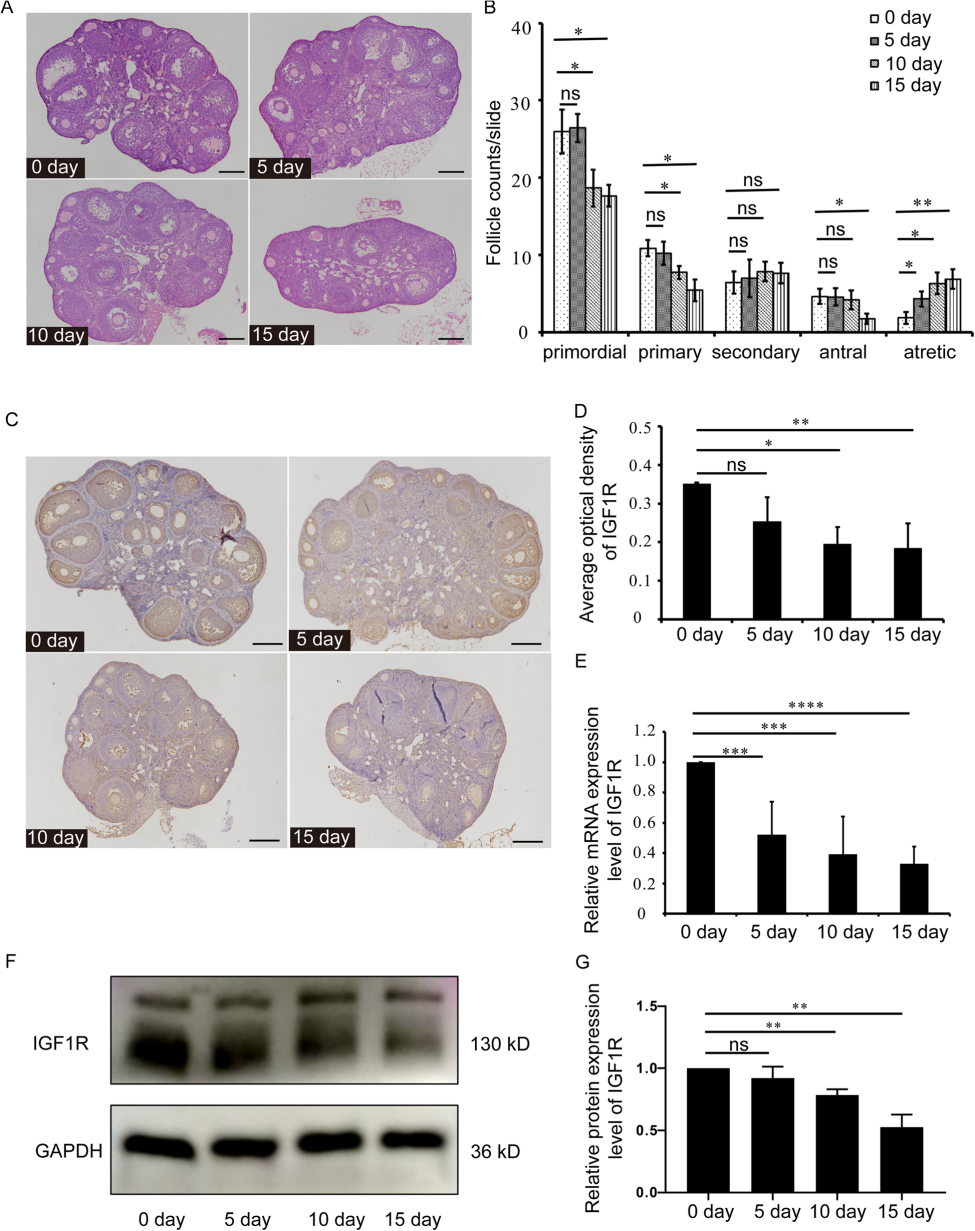
VCD down-regulated the expression of IGF1R in the ovarian GCs. **A** Histological observation of ovaries in mice which were intraperitoneally injected with VCD for 0, 5, 10 and 15 days respectively. Bar, 500 μm.** B** The counting results of different follicles, including primordial, primary, secondary, antral and atretic follicles (*n* = 9mice/group). **C** Representative images of IGF1R immunohistochemistry of ovary in mice which were intraperitoneally injected with VCD for 0, 5, 10 and 15 days respectively. Bar, 500 μm. **D** Quantitative analysis results of average optical density (AOD) of IGF1R in immunohistochemistry (*n* = 3mice/group). **E** Quantitative PCR results of IGF1R mRNA of ovary in mice which were intraperitoneally injected with VCD for 5, 10 and 15 days respectively (*n* = 9mice/group). **F** Western-blotting detection of IGF1R expression in ovary of mice which were intraperitoneally injected with VCD for 0, 5, 10 and 15 days respectively. **G** Quantitative analysis results of Western-blotting detection of IGF1R expression in ovaries(*n* = 3 mice/group). Each experiment was independently repeated at least three times. The results are expressed as means ± SEM. ns *p* > 0.05, *p < 0.05, **p < 0.01, ***p < 0.001, ****p < 0.0001

VCD was intraperitoneally injected into 6-week-old female C57BL/6 mice for 15 consecutive days. During this period, the ovaries were collected on day 5, day 10 and day 15 respectively, and HE staining was used to evaluate the effect of VCD on the development of follicles in the ovary (Fig. 2 A). As shown in Fig. 2 B, for primordial and primary follicles, after VCD injection for 5 days, there were no significant difference compared to day 0 group (*p* > 0.05). However, after VCD injection for 10 and 15 days, the counts of primordial and primary follicles were significantly reduced compared to the day 0 group (*p* < 0.05). For secondary follicles, VCD injection did not affect this stage follicles (*p* > 0.05). For antral follicles, the number of antral follicles decreased significantly compared to the day 0 group until 15 days after VCD injection (*p* < 0.05). In addition to the decline in counts of early-stage follicles, after VCD injection for 5, 10 and 15 days, the number of atretic follicles was significantly higher than that in the day 0 group (day 5 group and day 10 group: *p* < 0.05; day 15 group: *p* < 0.01) (Fig. 2 B). Meanwhile, the ovaries were collected on day 5, day 10 and day 15 of VCD injection respectively. The immunohistochemical results showed that IGF1R was mainly expressed in GCs of follicles at different developmental stages (Fig. 2 C). In day 0 and day 5 groups, strong immunoreactivity could be observed. But in day 10 and day 15 groups, there was only weak immunoreactivity (Fig. 2 C). The negative control was shown in Fig S3. The average optical density (AOD) of IGF1R in day 10 and day 15 groups was significantly decreased compared to day 0 group (day 10 group:* p* < 0.05; day 15 group: *p* < 0.01) (Fig. 2 D). Quantitative PCR further confirmed the above results. With the injection of VCD, the expression levels of *IGF1R* mRNA were gradually down-regulated compared to day 0 group (*p* < 0.001) (Fig. 2 E). Similar to the immunohistochemical results, Western-blotting analysis also showed that there was no significant difference in IGF1R protein expression between the day 0 and day 5 groups (*p* > 0.05). However, after VCD injection for 10 and 15 days, the IGF1R protein was significantly decreased compared to day 0 group (day 10 group and day 15 group: *p* < 0.01) (Fig. 2 F, G).

Since VCD mainly targeted the early-stage follicles, the ovaries of 6-day-old mice (mainly including the early-stage follicles) were cultured in vitro and treated with 0, 100, 250, 500, 750 and 1000 μM VCD for 48 h. Western-blotting results showed that there was no significant difference in IGF1R expression level between the 0 and 100 μM groups (*p* > 0.05) (Fig S4 A, B). But the IGF1R expression in 250, 500, 750, 1000 μM groups was significantly decreased compared to 0 μM group (250 μM group: *p* < 0.01; 500, 750 μM groups: *p* < 0.001; 1000 μM group: *p* < 0.0001) (Fig S4 A, B). The immunohistochemical results further confirmed that the expression of IGF1R in GCs was significantly reduced by 250 μM VCD treatment (*p* < 0.05) (Fig S4 C, D). Accordingly, follicle count results showed that 250 μM VCD treatment significantly decreased the number of the early-stage follicles in ovaries cultured in vitro (*p* < 0.01) (Fig S4 E, F). These results suggested that VCD could down-regulate the expression of IGF1R in the ovarian GCs.

### VCD inhibited the IGF1R/mTOR/AKT signaling pathway and induced autophagy in ovaries

**Fig. 3:**
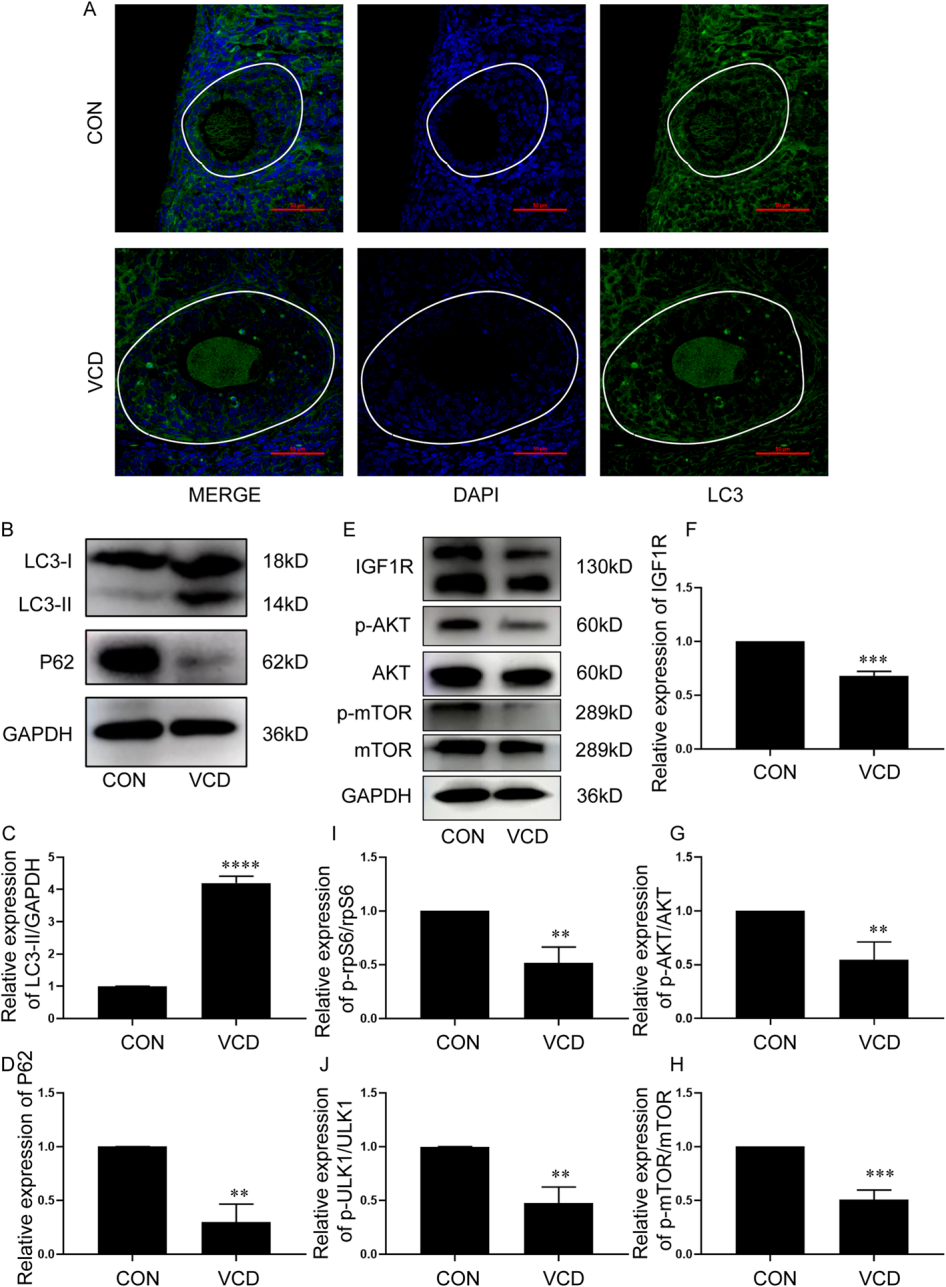
VCD inhibited the IGF1R/mTOR/AKT signaling pathway and induced autophagy in ovaries. **A** VCD was intraperitoneally injected into 6-week-old female C57BL/6 mice for 5 consecutive days (160 mg/kg per day), and the ovaries were collected 6 days after VCD administration. Immunofluorescence staining for LC3 (green); DAPI (blue) to visualize the nucleus. Arrow: Positive granulosa cell, White bracket: Follicles. Bar, 50 μm. **B** Western-blotting detection of p62, LC3-II expression in the ovaries. **C** Quantitative analysis results of Western-blotting detection of p62 expression in the ovaries (*n* = 3mice/group). **D** Quantitative analysis results of Western-blotting detection of LC3-II expression in the ovaries (*n* = 3mice/group). **E** Western-blotting detection of IGF1R, p-AKT/AKT, p-mTOR/mTOR expression in the ovaries. **F** Quantitative analysis results of Western-blotting detection of IGF1R expression in the ovaries(*n* = 3mice/group). **G** Quantitative analysis results of Western-blotting detection of p-AKT/AKT expression in the ovaries(*n* = 3mice/group). **H** Quantitative analysis results of Western-blotting detection of p-mTOR/mTOR expression in the ovaries (*n* = 3mice/group). Western-blotting detection of p-ULK1/ULK1 and p-rpS6/rpS6 expression in the ovaries, which was shown in Figure S10 A. **I** Quantitative analysis results of Western-blotting detection of p-ULK1/ULK1 expression in the ovaries(*n* = 3mice/group). **J** Quantitative analysis results of Western-blotting detection of p-rpS6/rpS6 expression in the ovaries (*n* = 3mice/group). Each experiment was independently repeated at least three times. The results are expressed as means ± SEM. **p < 0.01, ***p < 0.001, ****p < 0.0001. The control group was intraperitoneally injected with the same amount of sesame oil

VCD was intraperitoneally injected into 6-week-old female C57BL/6 mice for 5 consecutive days, the immunofluorescence results showed that puncta LC3 fluorescent signals were mainly observed in GCs of follicles in VCD treatment group (Fig. 3 A). Western-blotting analysis showed that VCD up-regulated autophagy markers LC3-II (*p* < 0.0001) and down-regulated P62 (*p* < 0.01) in ovaries (Fig. 3 B, C, D). The IGF1R/AKT/mTOR signaling pathway was detected, and the results showed that the IGF1R (*p* < 0.001) and its downstream signaling molecules p-AKT (*p* < 0.01) and p-mTOR(*p* < 0.001) were significantly decreased in the VCD treatment group compared with the control group (Fig. 3 E, F, G, H). The rpS6 is a well-known downstream target of mTOR, the reduction in p-rpS6 (*p* < 0.01) reflected the inhibition of mTOR and the induction of autophagy by VCD treatment (Figure S10 A; Fig. 3 I). Furthermore, we observed that VCD-induced phosphorylation of ULK1 at Ser757 in the ovary was significantly inhibited (*p* < 0.01) (Figure S10 A; Fig. 3 J). Collectively, the results suggested that VCD inhibited the IGF1R/mTOR/AKT signaling pathway and induced autophagy in ovaries.

### VCD inhibited the IGF1R/mTOR/AKT signaling pathway and induced autophagy in KGN cells

**Fig. 4:**
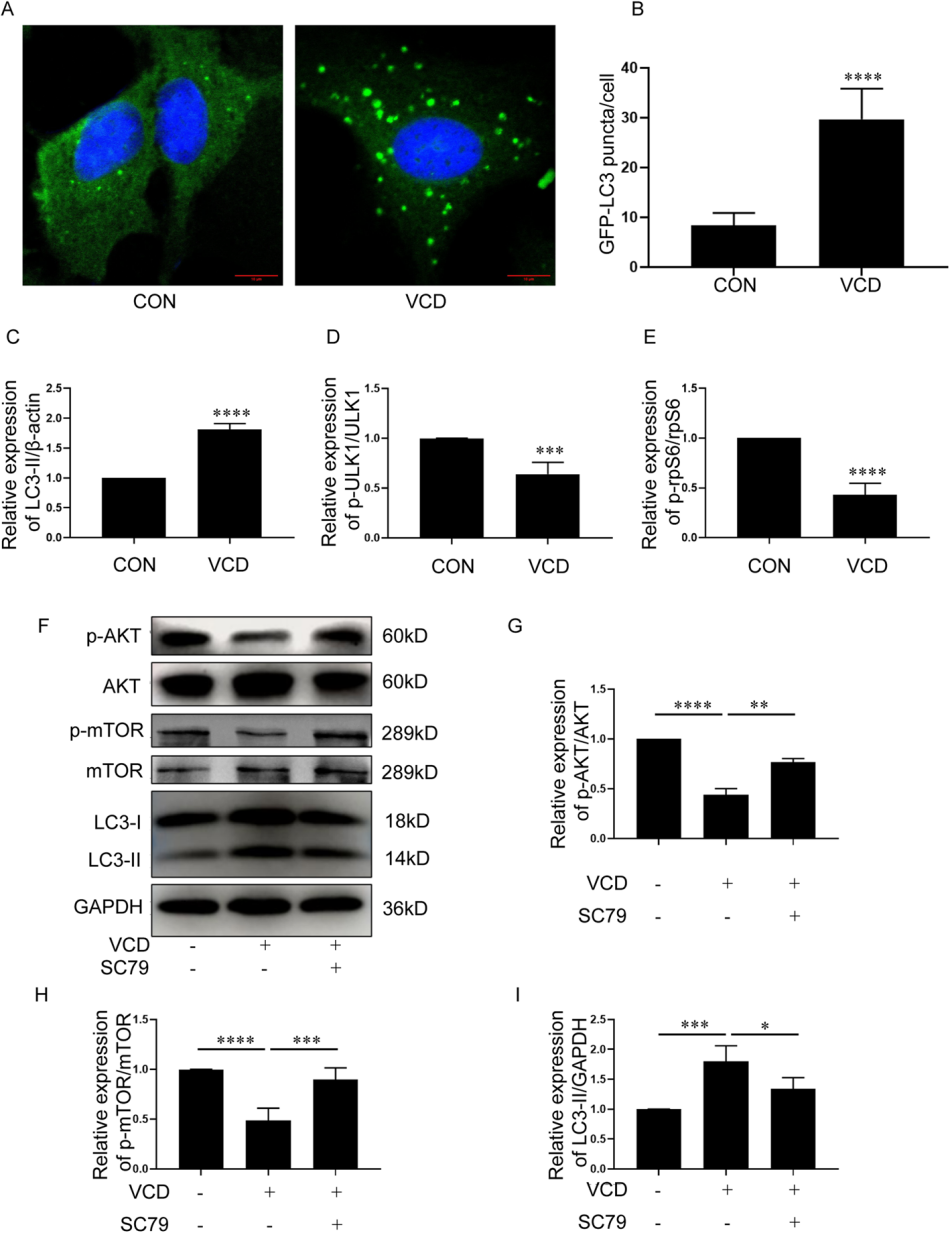
VCD inhibited the IGF1R/mTOR/AKT signaling pathway and induced autophagy in KGN cells. **A** GFP-LC3-KGN cells were treated with 0 (control) or 500 μM VCD for 24 h and detected by the confocal laser scanning microscope. Bar, 10 μm. **B** The counting results of the number of GFP-LC3 puncta per cell in KGN cells treated with 0 (control, *n* = 22)) or 500 μM VCD(*n* = 18) for 24 h. Western-blotting detection of LC3-II, p-ULK1/ULK1 and p-rpS6/rpS6 expression in KGN cells were treated with 0 (control) or 500 μM VCD for 24 h, which was shown in Figure S10 B. **C** Quantitative analysis results of Western-blotting detection of LC3-II expression in KGN cells were treated with 0 (control) or 500 μM VCD for 24 h(*n* = 5/group). **D** Quantitative analysis results of Western-blotting detection of p-ULK1/ULK1 expression in KGN cells were treated with 0 (control) or 500 μM VCD for 24 h(*n* = 4/group). **E** Quantitative analysis results of Western-blotting detection of p-rpS6/rpS6 expression in KGN cells were treated with 0 (control) or 500 μM VCD for 24 h(*n* = 5/group). **F** Western-blotting detection of p-AKT/AKT, p-mTOR/mTOR and LC3-II in KGN cells treated with 500 μM VCD alone or with 500 μM VCD and 10 μM SC79 for 24 h. (**G**) Quantitative analysis results of Western-blotting detection of p-AKT/AKT in KGN cells treated with 500 μM VCD alone or with 500 μM VCD and 10 μM SC79 for 24 h(*n* = 3/group). **H** Quantitative analysis results of Western-blotting detection of p-mTOR/mTOR in KGN cells treated with 500 μM VCD alone or with 500 μM VCD and 10 μM SC79 for 24 h(*n* = 5/group). **I** Quantitative analysis results of Western-blotting detection of LC3-II in KGN cells treated with 500 μM VCD alone or with 500 μM VCD and 10 μM SC79 for 24 h(*n* = 5/group). Each experiment was independently repeated at least three times. The results are expressed as means ± SEM. *p < 0.05, **p < 0.01, ***p < 0.001, ****p < 0.0001. The control group was treated with an equal volume of DMSO

KGN cells were treated with 0, 1, 10, 100, 500, 1000 and 1500 μM VCD for 24 h respectively. The cell viability was decreased when KGN cells were treated with 1000 and 1500 μM VCD for 24 h (Fig S5 A). Meanwhile, VCD could dose-dependently decrease the expression of IGF1R in KGN cells (Fig S5 B, C). Based on the above two results, 500 μM VCD was selected to be used in following studies. In order to confirm that the down-regulation of IGF1R expression is specifically caused by VCD rather than the simple cytotoxicity. Cyclophosphamide(CTX), another reagent commonly used to induce POF, was detected for its influence on the expression of IGF1R in KGN cells. For different concentrations of CTX, the expression of IGF1R in KGN cells was not affected regardless of whether it had an effect on the cell viability (Fig S5 D, E, F). The GFP-LC3-KGN cells were treated with 500 μM VCD for 24 h. The number of GFP-LC3 puncta in VCD-treated cells was significantly higher than that in control cells (*p* < 0.0001) (Fig. 4 A, B). In parallel, the expression level of LC3-II was increased obviously in VCD treatment cells compared to control cells (*p* < 0.0001) (Figure S10 B; Fig. 4C). And the phosphorylation of ULK1 (*p* < 0.001) and rp-S6 (*p* < 0.0001) were significantly reduced in VCD-treated cells compared to control cells (Figure S10 B; Fig. 4 D, E). These results suggested that autophagy in KGN cells was activated after VCD treatment. Whether the IGF1R/AKT/mTOR signaling pathway was involved in autophagy activation in VCD-treated KGN cells? Like in vivo results, VCD treatment decreased the phosphorylation of AKT (*p* < 0.0001) and mTOR (*p* < 0.0001) in KGN cells (Fig. 4 F, G, H). However, if the KGN cells were co-treated with VCD and AKT activator SC79, with an increase in the phosphorylation of AKT (*p* < 0.01) and mTOR (*p* < 0.001), the expression level of LC3-II was reduced obviously compared to VCD alone treatment (*p* < 0.05) (Fig. 4 F, G, H, I). These results indicated that VCD could induce autophagy by inhibiting the IGF1R/AKT/mTOR pathway in KGN cells.

### IGF1R knockdown induced autophagy in KGN cells

**Fig. 5:**
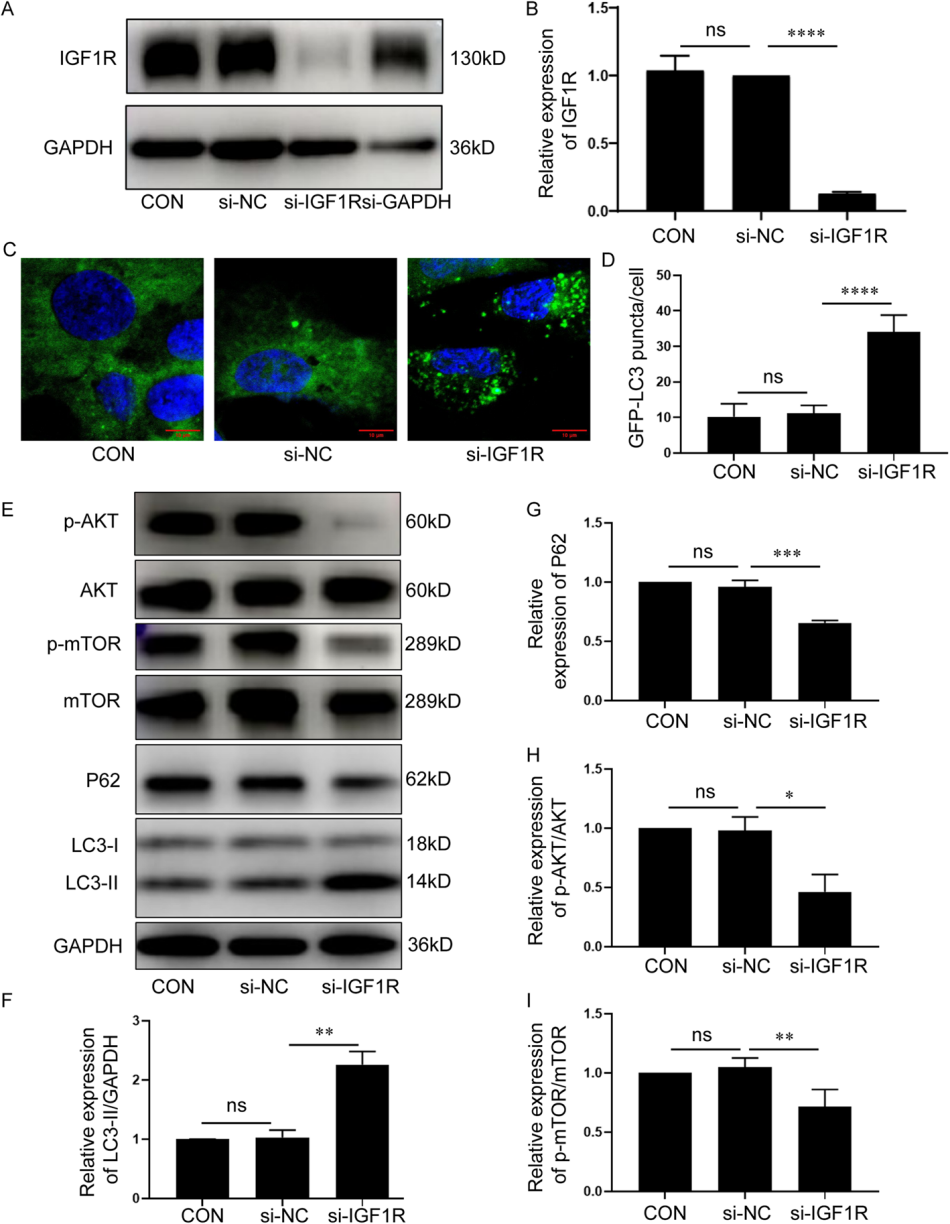
IGF1R knockdown induced autophagy in KGN cells. **A** Western-blotting detection of IGF1R expression in KGN cells which were transfected with 0(control), 50 nM *si-NC*, 50 nM *si-IGF1R* and 50 nM *si-GAPDH*. **B** Quantitative analysis results of Western-blotting detection of IGF1R expression in KGN cells which were transfected with 0(control), 50 nM *si-NC*, 50 nM *si-IGF1R* and 50 nM *si-GAPDH* (*n* = 3/group). **C** GFP-LC3-KGN cells were transfected with 0(control), 50 nM *si-NC*, 50 nM *si-IGF1R* and detected by the confocal laser scanning microscope. Bar, 10 μm. **D** The counting results of the number of GFP-LC3 puncta per cell in KGN cells transfected with 0(control), 50 nM *si-NC*, 50 nM *si-IGF1R* (*n* = 23,28,15/group). **E** Western-blotting detection of p-AKT/AKT, p-mTOR/mTOR, p62 and LC3-II in KGN cells transfected with 0(control), 50 nM *si-NC*, 50 nM *si-IGF1R*. **F** Quantitative analysis results of Western-blotting detection of LC3-II expression in KGN cells which were transfected with 0(control), 50 nM *si-NC*, 50 nM *si-IGF1R*(*n* = 3/group). (**G**) Quantitative analysis results of Western-blotting detection of p62 expression in KGN cells which were transfected with 0(control), 50 nM *si-NC*, 50 nM *si-IGF1R*(*n* = 3/group). **H** Quantitative analysis results of Western-blotting detection of p-AKT/AKT expression in KGN cells which were transfected with 0(control), 50 nM *si-NC*, 50 nM *si-IGF1R*(*n* = 5/group). **I** Quantitative analysis results of Western-blotting detection of p-mTOR/mTOR expression in KGN cells which were transfected with 0(control), 50 nM *si-NC*, 50 nM *si-IGF1R*(*n* = 5/group). Each experiment was independently repeated at least three times. The results are expressed as means ± SEM. ns *p* > 0.05, **p < 0.01, ***p < 0.001, ****p < 0.0001 vs *si-NC*. The control group was treated with DMEM/F12 medium supplemented with 10% FBS

Based on the results above, which showed that VCD down-regulated the expression of IGF1R in ovarian GCs, it is supposed that the inhibition of IGF1R/AKT/mTOR signal pathway induced by the down-regulation of IGF1R maybe the main reason for VCD-induced autophagy in ovarian GCs. The expression of IGF1R in KGN cells was knocked down by RNAi technology. Among the three synthesized small interfering RNAs, *si-IGF1R-2* had the highest interference efficiency and was used for subsequent experiments (Fig S6 A, B and Fig. 5 A, B). The GFP-LC3-KGN cells were transfected with 50 nM *si-IGF1R-2*, the number of GFP-LC3 puncta in *si-IGF1R* group was significantly increased compared with *si-NC* group (*p* < 0.0001), but there was no significant difference between control and *si-NC* groups (*p* > 0.05) (Fig. 5 C, D). Western-blotting results showed that compared with control and *si-NC* groups, the expression level of LC3-II was up-regulated (*p* < 0.01) and that of P62 was down-regulated (*p* < 0.001) after transfection with *si-IGF1R* (Fig. 5 E, F, G). Meanwhile, knockdown of IGF1R decreased the phosphorylation of AKT (*p* < 0.05) and mTOR (*p* < 0.01) in KGN cells, there was no significant difference between control and *si-NC* groups (*p* > 0.05) (Fig. 5 E, H, I). In order to further confirm the regulatory effect of IGF1R on autophagy, KGN cells were co-treated with SC79 following the knockdown of IGF1R (Fig S6 C, D). The phosphorylation of AKT (*p* < 0.001) and mTOR (*p* < 0.01) were increased, and the expression of LC3-II was reduced obviously compared to si-IGF1R alone treatment (*p* < 0.01) (Fig S6 C, E, F, G). These results indicated that consistent with VCD treatment, the inhibition of IGF1R/AKT/mTOR signal pathway, which was induced by the IGF1R knockdown, activated autophagy in ovarian GCs.

### VCD activated autophagy flux in KGN cells

Autophagy is a highly dynamic and multi-step process. Above results indicated that VCD could promote autophagosome accumulation, which may result from the acceleration of autophagosome formation or the inhibition of fusion between autophagosomes and lysosomes. Therefore, we used Bafilomycin A1 (Baf.A1), an inhibitor which could block the fusion of autophagosomes and lysosomes, to detect autophagy flux. The GFP-LC3-KGN cells were treated with VCD in combination with Baf.A1 or VCD alone, then labeled with the late lysosome marker LAMP-1 to detect its colocalization with the autophagosome and quantified the colocalization with Pearson correlation coefficient (PCC). If the autophagy flux is blocked, the colocalization of autophagosomes and lysosomes will decrease, in contrast, the autophagy flux is activated (Molero-Valenzuela et al. 2022; Sun et al. 2012; Tu et al. 2019; Zhong et al. 2022). The results showed that VCD alone treatment induced the GFP-LC3 punctation (*p* < 0.0001) and the colocalization of autophagosomes and lysosomes (*p* < 0.0001) compared to the control group (Fig S7 A, B, C). If the cells were treated with Baf.A1, whether co-treated with VCD or not, the GFP-LC3 punctation was significantly increased compared with control group (*p* < 0.0001), but little overlapping between autophagosomes and lysosomes was observed (Fig S7 A, B, C). Compared with the Baf.A1 alone group, VCD co-treated with Baf.A1 group further enhanced the punctation of GFP-LC3 (*p* < 0.0001), however, the colocalization of autophagosomes and lysosomes was not promoted (*p* < 0.05) (Fig S7 A, B, C). In parallel, the expression of the LC3-II in VCD co-treated with Baf.A1group was significantly up-regulated compared with VCD alone group (*p* < 0.0001) or Baf.A1 alone group (*p* < 0.05) (Fig S7 D, E). Similarly, if the expression of IGF1R was knockdown, the formation of GFP-LC3 puncta (*p* < 0.0001) and the colocalization with lysosome (*p* < 0.0001) increased significantly compared with *si-NC* group, but there was no significant difference between *si-IGF1R* group and VCD treatment group (Fig S7 F, G, H). These results demonstrated that VCD could down-regulate the expression of IGF1R and induce a high autophagy flux in KGN cells.

### VCD activated autophagy flux by regulating autophagy-related genes in KGN cells

**Fig. 6:**
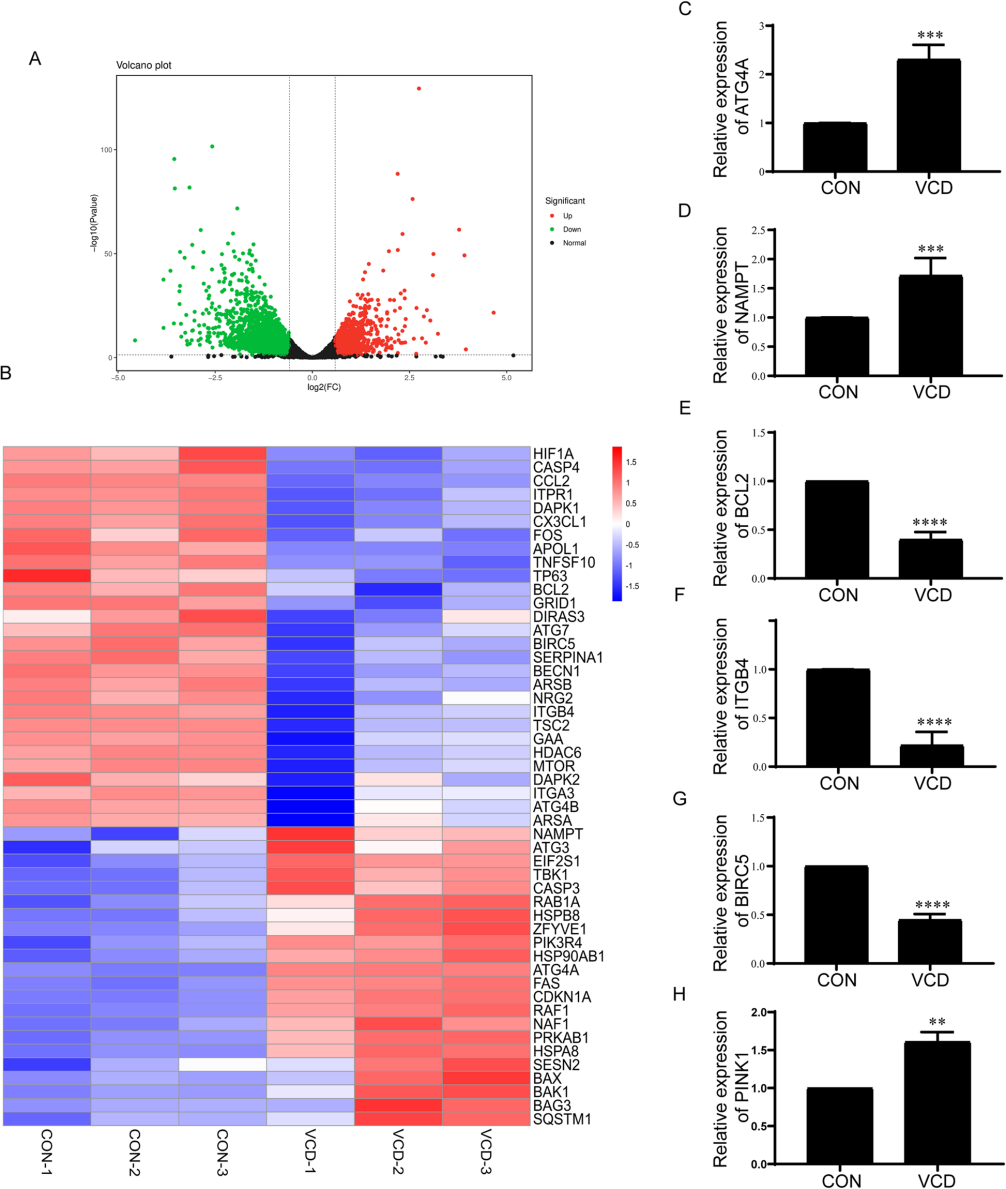
VCD activated autophagy flux by regulating autophagy-related genes in KGN cells. KGN cells were treated with 0 (control) or 500 μM VCD for 24 h. **A** Volcano plot of differentially expressed genes (DEGs) in KGN cells between control and VCD groups. A total of 4000 DEGs were discovered, 1356 were upregulated and 2464 were downregulated. DEGs were chosen by DESeq2 with adjusted Fold Change ≥ 1.5 and *p*-value < 0.05. **B** Hierarchical clustering of autophagy-related DEGs in *Human Autophagy Database (HADb)* between control and VCD groups, red and blue indicated the up-regulated and down-regulated genes respectively. Western-blotting detection of NAMPT, ATG4A, PINK1, BCL2, ITGB4, BIRC5 expression in KGN cells was shown in Figure S10 C. **C** Quantitative analysis results of Western-blotting detection of ATG4A expression in KGN cells(*n* = 4/group). **D** Quantitative analysis results of Western-blotting detection of NAMPT expression in KGN cells(*n* = 5/group). **E** Quantitative analysis results of Western-blotting detection of BCL2 expression in KGN cells(*n* = 4/group). **F** Quantitative analysis results of Western-blotting detection of ITGB4 expression in KGN cells(*n* = 6/group). **G** Quantitative analysis results of Western-blotting detection of BIRC5 expression in KGN cells(*n* = 4/group). **H** Quantitative analysis results of Western-blotting detection of PINK1 expression in KGN cells(*n* = 4/group). Each experiment was independently repeated at least three times. The results are expressed as means ± SEM. **p < 0.01, ***p < 0.001, ****p < 0.0001. The control group was treated with an equal volume of DMSO

Transcriptome sequencing (RNA-Seq) analysis was performed on KGN cells treated with VCD or not (control group). The volcano map showed the differentially expressed genes (DEGs) between the control group and VCD group. Compared to control group, 1356 DEGs were upregulated and 2464 DEGs were downregulated (Fig. 6 A and Table S1). Among these DEGs, the expression of 222 autophagy-related genes obtained from the Human Autophagy Database (http://www.autophagy.lu/clustering/index.html) was further analyzed, and 50 autophagy related DEGs were identified, including 22 up-regulated genes and 28 down-regulated genes (Fig. 6 B and Table S2). According to their role in the autophagy and ovarian function, 5 genes were selected for further validation by Western blot (Figure S10 C). Nicotinamide phosphoribosyltransferase (NAMPT) and Autophagy Related 4 Homolog A (ATG4A) increased significantly (*p* < 0.001). B-cell lymphoma-2 (BCL2), Integrin β4 (ITGB4), Baculoviral IAP Repeat Containing 5 (BIRC5) (*p* < 0.0001) were downregulated in VCD group compared with control group (Figure S10 C; Fig. 6 C, D, E, F, G). Meanwhile, given the role of NAMPT on mitophagy(Chen et al. 2020; Oka et al. 2021), there is a hypothesis that VCD induced mitophagy in KGN cells. PINK1, a critical molecule in mitophagy, was significantly up-regulated in VCD group (*p* < 0.01) (Figure S10 C; Fig. 6 H).

### Excessive autophagy flux induced by VCD treatment caused functional abnormalities in GCs

**Fig. 7:**
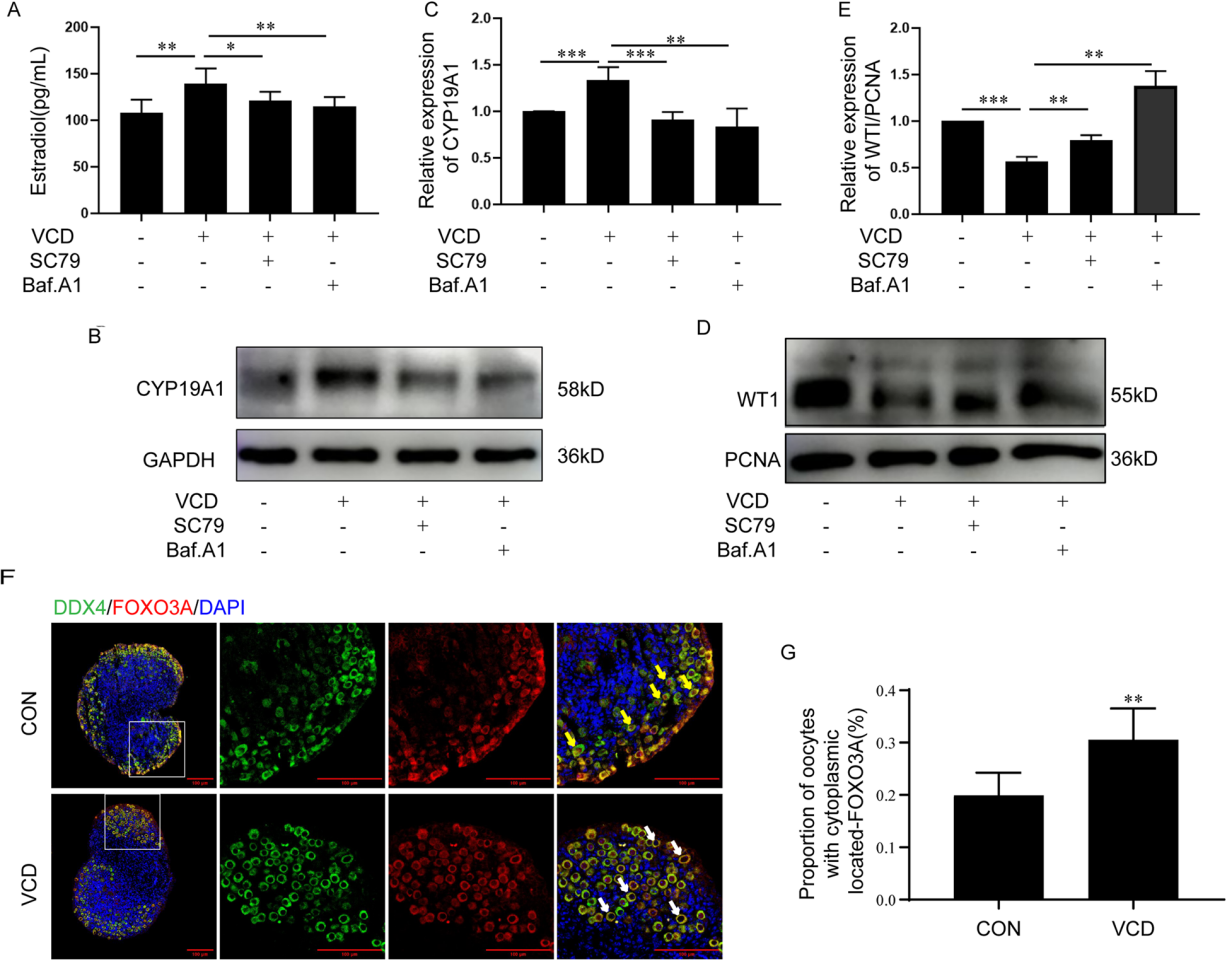
Excessive autophagy flux induced by VCD treatment caused functional abnormalities in GCs. (A) The E_2_ levels in cell culture supernatant of KGN cells treated with VCD (500 μM, 24 h) alone, VCD + SC79, and VCD + Baf.A1(*n* = 8,8,8,9/group). (B) Western-blotting detection of CYP19A1 in KGN cells treated with VCD (500 μM, 24 h) alone, VCD + SC79, and VCD + Baf.A1. (C) Quantitative analysis results of Western-blotting detection of CYP19A1 in KGN cells treated with VCD (500 μM, 24 h) alone, VCD + SC79, and VCD + Baf.A1(*n* = 5/group). (D) Western-blotting detection of WT1 in the nucleus of KGN cells treated with VCD (500 μM, 24 h) alone, VCD + SC79, and VCD + Baf.A1. (E) Quantitative analysis results of Western-blotting detection of WT1 in the nucleus of KGN cells treated with VCD (500 μM, 24 h) alone, VCD + SC79, and VCD + Baf.A1(*n* = 3/group). (F) Ovaries from 2-day-old female mice were cultured with or without 250 μM VCD for 24 h. Immunofluorescence results of FOXO3a (red), DDX4 (green) and DAPI (blue). Yellow arrows indicate dormant primordial follicles, white arrows indicate activated primordial follicles. Bar, 100 μm (left), 50 μm (enlarged view). (G) The proportion of cytoplasm localization of FOXO3a in the oocytes of ovaries from 2-day-old female mice cultured with or without 250 μM VCD for 24 h(*n* = 6/group). Each experiment was independently repeated at least three times. The results are expressed as means ± SEM. *p < 0.05, **p < 0.01, ***p < 0.001. The control group was treated with an equal volume of DMSO

To explore the effect of VCD-activated autophagy flux on GCs functions, the levels of E_2_ secreted by KGN cells treated with VCD (500 μM, 24 h) alone, VCD co-treated with SC79, and VCD co-treated with Baf.A1 were detected. Compared with control group, the level of E_2_ was significantly increased in the VCD alone group (*p* < 0.01) (Fig. 7 A). But, if AKT was activated by SC79 or the autophagy flux was blocked by Baf.A1, the levels of E_2_ were decreased compared with VCD alone group (VCD co-treated with SC79 group: *p* < 0.05; VCD co-treated with Baf.A1 group: *p* < 0.001) (Fig. 7 A). Accordingly, the expression level of CYP19A1, the rate-limiting enzyme for E_2_ synthesis, was up-regulated in VCD alone group compared with control group, VCD co-treated with SC79 group and VCD co-treated with Baf.A1 group (Fig. 7 B, C) (control group: *p* < 0.001; VCD co-treated with SC79: *p* < 0.001; VCD co-treated with Baf.A1: *p* < 0.01 vs VCD alone group). As the WT1 is an essential transcription factor for GCs, its selective degradation by the autophagy pathway is needed for the expression of steroidogenesis-related genes including CYP19A1 (Liu et al. 2023; Shao et al. 2022). Compared with the control group, the expression level of WT1 in nucleus was indeed reduced in VCD group (*p* < 0.001) (Fig. 7 D, E). However, if AKT was activated by SC79 or the autophagy flux was blocked by Baf.A1, the accumulation of WT1 was increased compared with VCD alone group (VCD co-treated with SC79 group: *p* < 0.01; VCD co-treated with Baf.A1 group: *p* < 0.01 vs VCD alone group) (Fig. 7 D, E). Based on the above results, it is suggested that the premature differentiation of GCs and the premature activation of primordial follicles are likely the reasons for ovarian follicle pool consumption induced by VCD. To evaluate the effect of VCD on primordial follicle development, ovaries from 2-day-old female mice were cultured in vitro with or without VCD for 24h and the expression of FOXO3a was detected. Expectably, VCD treatment enhanced the translocalization of FOXO3a from nucleus to cytoplasm in oocytes compared to the control group (*p* < 0.01) (Fig. 7 F, G). These results suggested that VCD induced WT1 selective degradation by the autophagy pathway leading to premature differentiation of GCs and the premature activation of primordial follicles.

## Discussion

The development of ovarian follicles is sensitive to many chemicals. VCD is a commonly used industrial chemicals which can lead to ovary toxicity. It is generally considered that VCD accelerates apoptosis in oocytes through the inhibition of AKT/mTOR signaling leading to follicle pool consumption (Chen et al. 2015; Kappeler & Hoyer 2012). In oocytes, the upstream regulator for Akt/mTOR is KITLG which is expressed by ovarian GCs. Losing the support function of GCs will cause oocyte apoptosis and further follicular atresia. Akt/mTOR activation is also crucial for function of ovarian GCs. Which is the upstream regulator for Akt/mTOR activation in ovarian GCs? Is it possible that this upstream regulator is a potential unknown target of VCD in ovarian GCs? In present study, IGF1R was found to be the potential target of VCD. VCD could down-regulate the expression of IGF1R in ovarian GCs in vivo and in vitro. KGN cells were considered to be close to immature granulosa cells (Nishi et al. 2001). Therefore, KGN cells can be used as an in vitro model of ovarian GCs in preantral follicles. Consistent with the results in ovaries, VCD also down regulated the expression of IGF1R in KGN cells. However, CTX, a chemotherapeutic drug which is also commonly used to induce POF (Qin et al. 2022), had no effect on IGF1R expression regardless of whether it had an effect on cell viability. This also suggests that VCD can specifically downregulate IGF1R expression in KGN cells, rather than due to cytotoxicity. Moreover, these results help to explain why the primordial and primary follicles are the selective targets of VCD. According to the theory of follicle development, follicle development can be classified into gonadotropin-independent phase, gonadotropin-responsive phase and gonadotropin-dependent phase, the primordial and primary follicles belong to gonadotropin-independent phase (Liu et al. 2019; Orisaka et al. 2021). In this phase, the survival and development of follicles are not dependent on FSH but are more dependent on intra-ovarian growth factors, especially the insulin-like growth factor system(Bezerra et al. 2018). Therefore, the primordial and primary follicles were more sensitive to the decrease in expression of IGF1R induced by VCD treatment.

Autophagy is an evolutionarily conserved process, autophagosomes sequestering cytoplasmic constituents, including damaged organelles and misfolded proteins, and subsequently fusing with lysosomes for degradation and recycling (Klionsky & Emr 2000; Levine & Klionsky 2004). As a key mechanism to regulate cellular homeostasis, autophagy is critical for regulating the follicular development during the whole reproductive lifespan. For instance, autophagy plays a vital role in overcoming the neonatal starvation to protect germ cells from excessive loss (Song et al. 2015) and regulating germ cell cyst breakdown (Wang et al. 2017). Nevertheless, excess autophagy also results in destruction of follicular reserve, which may lead to POF (Liu et al. 2023; Wu et al. 2018). As for ovarian GCs, the induction of autophagy is closely related to ovarian GCs apoptosis and ovarian follicular atresia at all stages of follicular development (Choi et al. 2011). However, as a commonly-used drug to induce POF and menopausal-related diseases in animal model, the effect of VCD on autophagy in ovary in still unknown. In present study, VCD led to an excessive accumulation of autophagosomes in ovarian GCs. Previous studies have also shown that autophagosomes were detected in ovarian GCs in the VCD-induced POF model (Dou et al. 2022; Qin et al. 2022). However, autophagosome accumulation may result from an increase in autophagosome formation or a decrease in the fusion with lysosomes for degradation. So that the effect of VCD on autophagy flux in GCs was further explored, the results indicated that VCD could activate a higher degree of autophagy flux in ovarian GCs. Consistently, VCD suppressed the phosphorylation of rpS6, reflecting the inhibition of mTOR and the initiation of autophagy (Saiki et al. 2011; Zhao et al. 2015). ULK1 is an essential kinase for autophagy, the activation of autophagy can be resulted from phosphorylation of Ser555 by AMPK or dephosphorylation of Ser757 by mTOR (Fan et al. 2017; Kim et al. 2011; Lee et al. 2020; Wong et al. 2015). Our results demonstrated that VCD led to a high degree of autophagy flux, which was dependent on the dephosphorylation of ULK1 at specific autophagy activation sites (Ser757).

The inhibition of IGF1R/AKT/mTOR signaling pathway induces autophagy in many cell types (Feng et al. 2022; Lum et al. 2005; Thiebaut et al. 2022; Wang et al. 2020). Both of VCD treatment and siRNA-mediated down-regulation of IGF1R expression resulted in the inhibition of the IGF1R/AKT/mTOR signaling pathway and the triggering of autophagy flux in KGN cells. RNA-Seq analysis further proved that VCD activated autophagy flux by up-regulating NAMPT, ATG4A and down-regulating BCL2, ITGB4, BIRC5. NAMPT and ATG4A stimulate autophagy flux by modulating AKT or mTOR signaling (Deng et al. 2022; Hsu et al. 2009; Wang et al. 2012; Yan et al. 2020), while BCL2, ITGB4, BIRC5 negatively regulate autophagy (Cheng et al. 2021; Ge et al. 2011; Lin et al. 2020; Liu et al. 2017; Sung et al. 2020; Thongchot et al. 2020; Yang et al. 2022; Zhang et al. 2020). Furthermore, NAMPT and BIRC5 have a pivotal role in ovarian functions. BIRC5 plays an important role in regulating folliculogenesis and oogenesis (Jiang et al. 2014). NAMPT promotes the secretion of E_2_ and the transcription of CYP19A1 (Reverchon et al. 2016; Thakre et al. 2021). Correspondingly, our results indeed indicated VCD treatment promoted the expression of CYP19A1 and the secretion of E_2_. Meanwhile, taking into account the positive effect of NAMPT on mitophagy (Chen et al. 2020; Oka et al. 2021), the impact of VCD on mitophagy in KGN cells was further investigated. VCD promoted the expression of PIKN1, indicating the activation of mitophagy. Recent studies indicate that excessive mitophagy is closely related to mitochondrial injury in ovarian GCs (Gannon et al. 2013; Yi et al. 2020). However, the mechanism of VCD-induced mitophagy remain to be further explored.

The production of E_2_ is an important indicator for the differentiation and functional maturation of ovarian GCs (Sugiura et al. 2010). In present study, VCD treatment promoted the expression of CYP19A1 and the secretion of E_2_. But, this promotive effect of VCD could be reversed by treatment with SC79 or Baf.A1, implying that VCD-induced excessive autophagy leads to premature differentiation of ovarian GCs. Autophagy may play a role in steroidogenesis (Gawriluk et al. 2014), inhibition of autophagy results in decreased expression of genes associated with ovarian GCs differentiation (Esmaeilian et al. 2023; Shao et al. 2022). It’s probably because autophagy mediates the degradation of the lipid droplets and total cholesterol to free cholesterol which is required for steroid synthesis (Esmaeilian et al. 2023; Ma et al. 2018). Interestingly, the WT1 is an essential transcription factor for GCs, its selective degradation by the autophagy pathway is needed for the expression of steroidogenesis-related genes and ovarian GCs differentiation (Liu et al. 2023; Shao et al. 2022). VCD treatment could reduce the accumulation of WT1 in nucleus. However, if AKT was activated by SC79 or the autophagy flux was blocked by Baf.A1, the accumulation of WT1 was increased. It is reported that the premature differentiation of ovarian GCs may induce the premature activation and rapid depletion of primordial follicles, eventually leading to POF (Cao et al. 2020; Zhang et al. 2022). Expectably, VCD treatment enhanced the translocalization of FOXO3a from nucleus to cytoplasm in oocytes of primordial follicles, indicating the premature activation of primordial follicles.

In summary, our study provides a novel mechanism about the ovarian toxicity of VCD which is attributed to the inhibition of IGF1R in granulosa cells, and the down-regulation of IGF1R induced excessive autophagy further led to granulosa cells dysfunction. This study sheds light on the role of autophagy in the functional regulation of granulosa cells, which not only reveals a new pathogenesis of POF but also suggests that maintaining autophagy homeostasis could be a strategy for treating POF. Further investigation is required to explore the autophagy-dependent degradation of WT1.

## Conclusion

In conclusion, the present study suggests a novel mechanism about the ovarian toxicity of VCD, whereby VCD inhibits IGF1R/AKT/mTOR signaling pathway by downregulating the expression of IGF1R and triggers excessive autophagy in ovarian GCs. Further, the excessive autophagy in ovarian GCs leads to the selective degradation of WT1, which promotes the expression of CYP19A1 and the secretion of E_2_, thus leading to the premature differentiation of ovarian GCs and the premature activation of primordial follicles resulting in the consumption of ovarian follicle pool. The Graphical Abstract was shown in Fig S8.

### Limitation

This study demonstrates that VCD specifically inhibits the expression of IGF1R in granulosa cells, but how VCD regulates IGF1R expression need to further investigate, specifically focusing on which proteins interact with or are involved in regulating IGF1R. Furthermore, this finding suggests that autophagy regulates the degradation of WT1, thereby impacting granulosa cell differentiation. Nevertheless, additional experiments are necessary to clarify the mechanisms of autophagy-mediated degradation of WT1.

## Materials and methods

### Animals and VCD treatment

C57BL/6 mice were purchased from the Laboratory Animal Center of Xi'an Jiaotong University and maintained under controlled conditions (22 °C, 50% humidity, 12 h light/dark cycle) with food and water available ad libitum. This study was approved by the Institutional Animal Ethical and Welfare Committee, Northwest A&F University, Shaanxi, China (Approval No. 2021065, Date. 2021.06.12). The chemical compound VCD (S40612-25 ml, Yuanye, Shanghai, China) was dissolved in sesame oil (S25527-500 mL, Yuanye, Shanghai, China) and intraperitoneally injected into 6-week-old female C57BL/6 mice with regular estrous cycles for 15 consecutive days (160 mg/kg per day) ( Shin et al. 2017; Li et al., 2020). The control mice were intraperitoneally injected with an equivalent amount of sesame oil. Each group contains 9 mice, samples were collected 45 days after VCD injection. The successful establishment of POF mouse model was confirmed by a disordered estrous cycle detected by vaginal smears after the injection of VCD. To further explore the mechanism of ovary toxicity induced by VCD, 6-week-old female C57BL/6 mice were intraperitoneally injected with VCD (160 mg/kg per day), ovaries were collected on 0 day, 5 days, 10 days and 15 days respectively after VCD injection, each group contains 9 mice.

### Cell culture and the establishment of GFP-LC3-KGN cell line

Human granulosa-like tumor cell line KGN cells were obtained from Professor Liu Dewu in the Herbivore Laboratory of South China Agricultural University. KGN cells were cultured in Ham's F-12 Nutrient Mixture and Dulbecco's Modified Eagles Medium (1:1, DMEM/F12) (SH30271.01, HyClone, Los Angeles, CA) containing 10% FBS (2,324,371, Gibco, New York, NY).

The GFP-LC3-KGN cell line was established by transfection with a GFP-LC3 lentivirus. Briefly, the 293T cells were cultured in 100mm dish with DMEM/F12 medium supplemented with 10% FBS, when the cells reached 90% confluence, 10 μg GFP-LC3 plasmids and 10 μg three packaging plasmids pLP/VSVG, pLP1 and pLP2 were co-transfected into 293T cells using Lipofectamine™ 3000 (L3000015, Invitrogen, Carlsbad, CA, USA). The medium containing GFP-LC3-lentivirus was harvested at 48h after co-transfection and concentrated with PEG. KGN cells were cultured in a 35 mm dish and infected with the GFP-LC3 lentivirus. After 48 h, the cells were screened with 2 μg/mL puromycin for 48 h. Establishment of GFP-LC3-KGN cell line was confirmed by observing GFP-LC3 puncta in the KGN cells incubated with DMEM/F12 medium without FBS (serum starvation) for 16h. The GFP-LC3 puncta in the cells were observed by confocal microscope (Nikon A1Rsi, Nikon, Tokyo, Japan) and the quantitative analysis of the number of GFP-LC3 puncta was shown in Fig S9.

For VCD treatment, cells were treated with 0.1, 1, 10, 100, 500 and 1000 μM VCD respectively for 24 h. To confirm that the downregulation of IGF1R expression was specifically caused by VCD, KGN cells were also treated with 0, 5 and 30 mg/L cyclophosphamide (CTX) (S30563, Yuanye, Shanghai, China) (another common chemical for POF molding).

### Ovarian culture in vitro and VCD treatment

To detect the IGF1R expression, ovaries from 6-day-old female C57BL/6 mice were harvested. To evaluate the effect of VCD on primordial follicle development, ovaries from 2-day-old female C57BL/6 mice were harvested. The ovaries were cultured in 8-μm transwell inserts under 37 °C with atmosphere containing 5% CO_2_. The ovarian culture medium was Dulbecco's Modified Eagles Medium (DMEM) (SH30022.01, HyClone, Los Angeles, CA) containing 10% Fetal Bovine Serum (FBS) (2,324,371, Gibco, New York, NY) and 0.5 IU/ml follicle-stimulating hormone (FSH) (HOR-294, Prospecbio, Israel). Ovaries were randomly assigned to receive different treatment: control group and VCD group (250 μM).

### Histological and immunohistochemical assessment

Ovaries were fixed with 4% paraformaldehyde (P0099-500 ml, Beyotime Institute of Biotechnology, Shanghai, China), dehydrated, and embedded in paraffin. A series of 5-μm sections were prepared and one of every five sections was selected for hematoxylin and eosin staining. Ovarian morphology was observed and photographed with an optical microscope (Nikon Ni-U, Japan). The average number of follicles in all stages of each slide was counted. The primordial follicle contains a small primary oocyte with a single layer of flat granulosa cells tightly attached to the oocyte. The primary follicle contains a single layer of cuboidal granulosa cells surrounded the oocyte. The oocytes of secondary follicles are surrounded by multiple layers granulosa cells, and the acquisition of a theca. The antral follicle is characterized by a cavity containing follicular fluid (Williams & Erickson 2012). The immunohistochemistry used the streptavidin-peroxidase method. Antigens were retrieved by boiling for 15 min in Citrate Antigen Retrieval solution (C1032, Solarbio, China). Endogenous peroxidase was blocked by incubation in 3% hydrogen peroxide. The primary antibody anti-IGF1R (Table S3) was incubated overnight at 4℃, and the secondary antibody was incubated at 37℃ for 1 h. Then the sections were lightly counterstained with hematoxylin, dehydrated and cover-slipped. The immunohistochemical results were analyzed quantitatively using Image J 1.8.0.

### TUNEL assay

According to the TUNEL kit (green fluorescence, C1088, Beyotime Institute of Biotechnology, Shanghai, China) manufacturer's instructions, sections of ovaries were deparaffinized and boiled in sodium citrate buffer at 95℃ for 20 min. After three rounds of antigen repair, the section was closed with 1% BSA. The TUNEL test solution was applied to sections at 37℃ for 60 min in the dark, and counterstained with DAPI, the images were captured using confocal microscope (Nikon, Tokyo, Japan).

### Immunofluorescence

As for the detection of autophagy flux, GFP-LC3-KGN were fixed with 4% paraformaldehyde for 30 min and incubated with 0.2% TritonX-100 (T8532-100 ml, Sigma-Aldrich, St. Louis, MO, USA) at 25 °C for 10 min. After washing, the cells were blocked with 1% BSA and then incubated with the primary antibody anti-LAMP1 at 4℃ overnight, and Alexa Flour 594 secondary antibodies at 37℃ for 1 h, images were captured with confocal microscope (Nikon, Tokyo, Japan). The Pearson coefficient of colocalization between 2 fluorophores was calculated using Image J 1.8.0. Cell nucleus was stained with DAPI (28718–90-3, Sigma, USA) at 25 °C for 10 min.

For tissue immunofluorescence, ovary sections were deparaffinized and antigen repaired for subsequent antigen retrieval. After blocking with BSA (S12012-100 g, Yuanye, Shanghai, China), slides were stained with primary antibody anti-LC3 and the secondary antibody coupled with Alexa-488 according to the above methods. The details of antibodies used for immunofluorescence is listed in Table S3.

### RNA interference (RNAi) of IGF1R

The small interfering RNA (siRNA) and negative control RNA (NC siRNA) of IGF1R were synthesized by Tsingke (Beijing, China). When the confluence of KGN and GFP-LC3-KGN reached 80–90%, siRNA(50 nmol/L) was transfected with LipofectamineTM 3000 (L3000015, Invitrogen, USA) for 48 h. The oligos for siRNA are listed in Table S4.

### RNA extraction, reverse transcription and quantitative real-time PCR

Total RNA was extracted in super clean bench using RNAiso Plus (9109, Takara Corporation, Dalian, China) according to the manufacturer’s instructions and the gDNA Eraser Spin Column and DNase I were used to remove gDNA during RNA extraction. RNA purity and concentration were assessed by microvolume spectrophotometer (NanoDrop One, Thermo Scientific). cDNA was synthesized using PrimeScript™ RT Reagent Kit (RR047A, Takara Corporation, Dalian, China). The primer sequences and accession number are shown in Table S5. The quantitative RT-PCR reactions were performed with the Fast SYBR Green Master Mix (04913914001–1, Genstar, Beijing, China), data collection and analysis were performed on the QuantStudio 6 Flex machine (Invitrogen Corporation, Waltham, MA) using the GraphPad Prism 6 software. The quantitative RT-PCR parameters were as follows: 95℃ for 2 min, followed by 40 cycles each of 95℃ for 15 s, 60℃ for 30 s, and 72℃ for 30 s. All samples run in triplicate reactions. Relative quantities of the amplified products were calculated according to the 2^−ΔΔCT^ method. Relative gene expression was obtained by normalizing with GAPDH expression, calculating the differences in mRNA expression as fold changes relative to the expression in the day 0 group.

### Western blot

To extract total protein, KGN cells were collected in High-efficiency RIPA buffer (R0010, Solarbio, Beijing, China) including protease and phosphatase inhibitors according to the manufacturer’s instructions. To separate nuclear protein, the KGN cells were seeded in 60-mm dishes and treated with SC79 or Baf.A1. The nuclear protein was separated according to the manufacturer’s protocol (PK10014, Proteintech, Chicago, IL, USA, PK10014). Equal amounts of proteins were used to SDS-PAGE, and then transferred to PVDF membranes. The 5% nonfat milk was applied to block the nonspecific binding. Membranes were incubated with the primary antibody overnight at 4 °C. The blots were then incubated with HRP-labeled donkey anti-mouse/rabbit IgG (H + L). Images were collected by enhanced chemiluminescence autoradiography (Biotanon, Shanghai, China). Normalization was performed by calculating the ratios to β-actin levels after quantification of the bands with Image J 1.8.0. The details of antibodies used for Western blot is listed in Table S3.

### Hormone measurement

For E_2_ detection, KGN cells were cultured with Phenol Red-free DMEM/F-12 (SH30272.01, HyClone, China), charcoal-stripped FBS (A1061, YOSHi TECHNOLOGY, Wuhan, China) and 10 nM testosterone (C6163A, APExBIO, Houston, USA). KGN cells were seeded in 24 well plates at a density of 1 × 10^5^ cells per well, when the density reached 80% KGN cells were treated with 0 μM (control), 500 μM VCD, 500 μM VCD co-treated with 10 μM SC79 for 24h, pretreated with Baf.A1 (0.1 μM) for 1 h and then treated with VCD (500 μM) for 24h. The supernatant was collected for E_2_ detection by ELISA Kit (E-OSEL-H0005, Elabscience, Wuhan, China).

### Transcriptome sequencing

KGN cells were treated with 500 μM VCD for 24 h, and the total RNA was extracted with RNAiso Plus (Takara Corporation, China). The concentration and integrity were examined respectively by Nanodrop2000 and Agilent 2100 bioanalyzer (Thermo Fisher Scientific, USA), the mRNA was purified and fragmented by VAHTS mRNA Capture Beads (Vazyme, China). The first and second-strand cDNA were synthesized, purified for terminal repair, amplified, and the quality was determined by Qsep-400. Single-strand circular cDNA was denatured, cycled and digested from double-stranded cDNA to construct the library and sequenced by Illumina HiSeq platform (Tsingke, Beijing, China). Differentially expressed genes (DEGs) were chosen by the DESeq2 with adjusted Fold Change ≥ 1.5 and *p*-value < 0.05.

### Statistical analysis

All data were expressed as the Mean ± SEM from at least three independent experiments. The sample size and replicates number for statistical analysis were labeled in figure legend. Statistical analysis was performed by GraphPad Prism 8.0.1 software (GraphPad, San Diego, CA, USA). The normality and lognormality tests were performed by GraphPad Prism 8.0.1 software. If the data were passed normality test(alpha = 0.05), differences between two groups were evaluated by the Student’s t-test. For more than two groups, one-way analysis of variance (ANOVA) followed by Dunnett’s multiple comparison tests were used, and for experimental set-ups with a second variable (follicle counting), two-way ANOVA followed by Dunnett’s multiple comparison tests were used. For non-normal data, differences among three or more independent groups were evaluated by Kruskal–Wallis. *P*-values were calculated, and statistical significance is displayed by asterisks (*p < 0.05, **p < 0.01, ***p < 0.001, ****p < 0.0001, ns: no significance, *p* > 0.05).

## Supplementary Information

Supplementary Material 1.

Supplementary Material 2.

## Data Availability

The accession number of transcriptome sequencing rawdata reported in this paper is NCBI: PRJNA1047516. And the data that support the findings of this study are available from the corresponding author upon reasonable request.

## Abbreviations

AKT: Protein kinase B
AMH: Anti-Müllerianhormone
ATG: Autophagy related
ATG4A: Autophagy Related 4 Homolog A
Baf.A1: Bafilomycin A1
BCL2: B-cell lymphoma-2
BIRC5: Baculoviral IAP Repeat Containing 5
CTX: Cyclophosphamide
E_2_: Estradiol
FSH: Follicle-stimulating hormone
GCs: Granulosa cells
IGF1R: Insulin-like growth factor 1 receptor
ITGB4: Integrin β4
KGN: Granulosa tumour cell line
LC3: Microtubule-associated protein 1 light chain 3
mTOR: Mammalian target of rapamycin
NAMPT: Nicotinamide phosphoribosyltransferase
PINK1: PTEN induced kinase 1
POF: Premature ovarian failure
siRNA: Small interfering RNA
VCD: 4-Vinylcyclohexene diepoxide
WT1: WT1 transcription factor

## References

[CR1] Baumgarten SC, Armouti M, Ko C, Stocco C (2017) IGF1R Expression in Ovarian Granulosa Cells Is Essential for Steroidogenesis, Follicle Survival, and Fertility in Female Mice. Endocrinology 158(7):2309–2318. 10.1210/en.2017-00146 28407051 10.1210/en.2017-00146 PMC5505221

[CR2] Bezerra MES, Barberino RS, Menezes VG, Gouveia BB, Macedo TJS, Santos JMS, Monte APO, Barros VRP, Matos MHT (2018) Insulin-like growth factor-1 (IGF-1) promotes primordial follicle growth and reduces DNA fragmentation through the phosphatidylinositol 3-kinase/protein kinase B (PI3K/AKT) signalling pathway. Reprod Fertil Dev 30(11):1503–1513. 10.1071/RD17332 29843892 10.1071/RD17332

[CR3] Bhardwaj JK, Paliwal A, Saraf P, Sachdeva SN (2022) Role of autophagy in follicular development and maintenance of primordial follicular pool in the ovary. J Cell Physiol 237(2):1157–1170. 10.1002/jcp.30613 34668576 10.1002/jcp.30613

[CR4] Cannady EA, Dyer CA, Christian PJ, Sipes IG, Hoyer PB (2003) Expression and activity of cytochromes P450 2E1, 2A, and 2B in the mouse ovary: the effect of 4-vinylcyclohexene and its diepoxide metabolite. Toxicol Sci 73(2):423–430. 10.1093/toxsci/kfg077 12700394 10.1093/toxsci/kfg077

[CR5] Cao LB, Leung CK, Law PW, Lv Y, Ng CH, Liu HB, Lu G, Ma JL, Chan WY (2020) Systemic changes in a mouse model of VCD-induced premature ovarian failure. Life Sci 262. 10.1016/j.lfs.2020.118543 10.1016/j.lfs.2020.118543 33038381

[CR6] Cao LB, Liu HB, Lu G, Lv Y, Leung CK, Du YZ, Wang WM, Xiong ZQ, Su XW, Li HJ, Chen ZJ, Ma JL, Chan WY (2020) Hormone-Like Effects of 4-Vinylcyclohexene Diepoxide on Follicular Development. Front Cell Dev Biol 8:587. 10.3389/fcell.2020.00587 32850784 10.3389/fcell.2020.00587 PMC7412635

[CR7] Castro-Cruz A, Echeverria OM, Sanchez-Sanchez L, Munoz-Velasco I, Juarez-Chavero S, Torres-Ramirez N, Vazquez-Nin GH, Escobar ML (2023) Dissection of the autophagic route in oocytes from atretic follicles. Biol Cell 115(3):e2200046. 10.1111/boc.202200046 36571578 10.1111/boc.202200046

[CR8] Chen Z, Kang X, Wang L, Dong H, Wang C, Xiong Z, Zhao W, Jia C, Lin J, Zhang W, Yuan W, Zhong M, Du H, Bai X (2015) Rictor/mTORC2 pathway in oocytes regulates folliculogenesis, and its inactivation causes premature ovarian failure. J Biol Chem 290(10):6387–6396. 10.1074/jbc.M114.605261 25564616 10.1074/jbc.M114.605261 PMC4358274

[CR9] Chen F, Feng L, Zheng YL, Lu J, Fan SH, Shan Q, Zheng GH, Wang YJ, Wu DM, Li MQ, Wang QQ, Zhang ZF (2020) 2, 2’, 4, 4’-tetrabromodiphenyl ether (BDE-47) induces mitochondrial dysfunction and related liver injury via eliciting miR-34a-5p-mediated mitophagy impairment. Environ Pollut 258:113693. 10.1016/j.envpol.2019.113693 31838391 10.1016/j.envpol.2019.113693

[CR10] Cheng SM, Lin TY, Chang YC, Lin IW, Leung E, Cheung CHA (2021) YM155 and BIRC5 downregulation induce genomic instability via autophagy-mediated ROS production and inhibition in DNA repair. Pharmacol Res 166:105474. 10.1016/j.phrs.2021.105474 33549731 10.1016/j.phrs.2021.105474

[CR11] Choi JY, Jo MW, Lee EY, Yoon BK, Choi DS (2010) The role of autophagy in follicular development and atresia in rat granulosa cells. Fertil Steril 93(8):2532–2537. 10.1016/j.fertnstert.2009.11.021 20149359 10.1016/j.fertnstert.2009.11.021

[CR12] Choi J, Jo M, Lee E, Choi D (2011) Induction of apoptotic cell death via accumulation of autophagosomes in rat granulosa cells. Fertil Steril 95(4):1482–1486. 10.1016/j.fertnstert.2010.06.006 20630503 10.1016/j.fertnstert.2010.06.006

[CR13] Deng Y, Duan R, Ding W, Gu Q, Liu M, Zhou J, Sun J, Zhu J (2022) Astrocyte-derived exosomal nicotinamide phosphoribosyltransferase (Nampt) ameliorates ischemic stroke injury by targeting AMPK/mTOR signaling to induce autophagy. Cell Death Dis 13(12):1057. 10.1038/s41419-022-05454-9 36539418 10.1038/s41419-022-05454-9 PMC9767935

[CR14] Denton D, Kumar S (2019) Autophagy-dependent cell death. Cell Death Differ 26(4):605–616. 10.1038/s41418-018-0252-y 30568239 10.1038/s41418-018-0252-y PMC6460387

[CR15] Dou X, Jin X, Chen X, Zhou Q, Chen H, Wen M, Chen W (2022) Bu-Shen-Ning-Xin decoction alleviates premature ovarian insufficiency (POI) by regulating autophagy of granule cells through activating PI3K/AKT/mTOR pathway. Gynecol Endocrinol 38(9):754–764. 10.1080/09513590.2022.2112941 35989579 10.1080/09513590.2022.2112941

[CR16] Esmaeilian Y, Hela F, Bildik G, Iltumur E, Yusufoglu S, Yildiz CS, Yakin K, Kordan Y, Oktem O (2023) Autophagy regulates sex steroid hormone synthesis through lysosomal degradation of lipid droplets in human ovary and testis. Cell Death Dis 14(5):342. 10.1038/s41419-023-05864-3 37236920 10.1038/s41419-023-05864-3 PMC10220221

[CR17] Fan Y, Wang N, Rocchi A, Zhang W, Vassar R, Zhou Y, He C (2017) Identification of natural products with neuronal and metabolic benefits through autophagy induction. Autophagy 13(1):41–56. 10.1080/15548627.2016.1240855 27791467 10.1080/15548627.2016.1240855 PMC5240827

[CR18] Feng L, Li B, Xi Y, Cai M, Tian Z (2022) Aerobic exercise and resistance exercise alleviate skeletal muscle atrophy through IGF-1/IGF-1R-PI3K/Akt pathway in mice with myocardial infarction. Am J Physiol Cell Physiol 322(2):C164–C176. 10.1152/ajpcell.00344.2021 34852207 10.1152/ajpcell.00344.2021

[CR19] Gannon AM, Stampfli MR, Foster WG (2013) Cigarette smoke exposure elicits increased autophagy and dysregulation of mitochondrial dynamics in murine granulosa cells. Biol Reprod 88(3):63. 10.1095/biolreprod.112.106617 23325812 10.1095/biolreprod.112.106617

[CR20] Gannon OJ, Naik JS, Riccio D, Mansour FM, Abi-Ghanem C, Salinero AE, Kelly RD, Brooks HL, Zuloaga KL (2023) Menopause causes metabolic and cognitive impairments in a chronic cerebral hypoperfusion model of vascular contributions to cognitive impairment and dementia. Biol Sex Differ 14(1):34. 10.1186/s13293-023-00518-7 37221553 10.1186/s13293-023-00518-7 PMC10204285

[CR21] Gawriluk TR, Hale AN, Flaws JA, Dillon CP, Green DR, Rucker EBR (2011) Autophagy is a cell survival program for female germ cells in the murine ovary. Reproduction 141(6):759–765. 10.1530/REP-10-0489 21464117 10.1530/REP-10-0489

[CR22] Gawriluk TR, Ko C, Hong X, Christenson LK, Rucker EBR (2014) Beclin-1 deficiency in the murine ovary results in the reduction of progesterone production to promote preterm labor. Proc Natl Acad Sci U S A 111(40):E4194-4203. 10.1073/pnas.1409323111 25246579 10.1073/pnas.1409323111 PMC4210046

[CR23] Ge D, Jing Q, Meng N, Su L, Zhang Y, Zhang S, Miao J, Zhao J (2011) Regulation of apoptosis and autophagy by sphingosylphosphorylcholine in vascular endothelial cells. J Cell Physiol 226(11):2827–2833. 10.1002/jcp.22632 21302284 10.1002/jcp.22632

[CR24] He T, Liu Y, Zhao S, Liu H, Wang Z, Shi Y (2019) Comprehensive assessment the expression of core elements related to IGFIR/PI3K pathway in granulosa cells of women with polycystic ovary syndrome. Eur J Obstet Gynecol Reprod Biol 233:134–140. 10.1016/j.ejogrb.2018.12.010 30594023 10.1016/j.ejogrb.2018.12.010

[CR25] Hoyer PB, Sipes IG (2007) Development of an animal model for ovotoxicity using 4-vinylcyclohexene: a case study. Birth Defects Res B Dev Reprod Toxicol 80(2):113–125. 10.1002/bdrb.20103 17342769 10.1002/bdrb.20103

[CR26] Hsu CP, Oka S, Shao D, Hariharan N, Sadoshima J (2009) Nicotinamide phosphoribosyltransferase regulates cell survival through NAD+ synthesis in cardiac myocytes. Circ Res 105(5):481–491. 10.1161/CIRCRESAHA.109.203703 19661458 10.1161/CIRCRESAHA.109.203703 PMC2765790

[CR27] Hu X, Christian PJ, Thompson KE, Sipes IG, Hoyer PB (2001) Apoptosis induced in rats by 4-vinylcyclohexene diepoxide is associated with activation of the caspase cascades. Biol Reprod 65(1):87–93. 10.1095/biolreprod65.1.87 11420227 10.1095/biolreprod65.1.87

[CR28] Hubbard EF, Mashouri P, Pyle WG, Power GA (2023) The effect of gradual ovarian failure on dynamic muscle function and the role of high-intensity interval training on mitigating impairments. Am J Physiol Cell Physiol 325(4):C1031–C1045. 10.1152/ajpcell.00318.2023 37661923 10.1152/ajpcell.00318.2023

[CR29] Jiang ZZ, Hu MW, Wang ZB, Huang L, Lin F, Qi ST, Ouyang YC, Fan HY, Schatten H, Mak TW, Sun QY (2014) Survivin is essential for fertile egg production and female fertility in mice. Cell Death Dis 5(3):e1154. 10.1038/cddis.2014.126 24675472 10.1038/cddis.2014.126 PMC3973204

[CR30] Jiao W, Mi X, Yang Y, Liu R, Liu Q, Yan T, Chen ZJ, Qin Y, Zhao S (2022) Mesenchymal stem cells combined with autocrosslinked hyaluronic acid improve mouse ovarian function by activating the PI3K-AKT pathway in a paracrine manner. Stem Cell Res Ther 13(1):49. 10.1186/s13287-022-02724-3 35109928 10.1186/s13287-022-02724-3 PMC8812195

[CR31] Kappeler CJ, Hoyer PB (2012) 4-vinylcyclohexene diepoxide: a model chemical for ovotoxicity. Syst Biol Reprod Med 58(1):57–62. 10.3109/19396368.2011.648820 22239082 10.3109/19396368.2011.648820 PMC3307534

[CR32] Kim J, Kundu M, Viollet B, Guan KL (2011) AMPK and mTOR regulate autophagy through direct phosphorylation of Ulk1. Nat Cell Biol 13(2):132–141. 10.1038/ncb2152 21258367 10.1038/ncb2152 PMC3987946

[CR33] Klionsky DJ, Emr SD (2000) Autophagy as a regulated pathway of cellular degradation. Science 290(5497):1717–1721. 10.1126/science.290.5497.1717 11099404 10.1126/science.290.5497.1717 PMC2732363

[CR34] Konhilas JP, Sanchez JN, Regan JA, Constantopoulos E, Lopez-Pier M, Cannon DK, Skaria R, McKee LA, Chen H, Lipovka Y, Pollow D, Brooks HL (2020) Using 4-vinylcyclohexene diepoxide as a model of menopause for cardiovascular disease. Am J Physiol Heart Circ Physiol 318(6):H1461–H1473. 10.1152/ajpheart.00555.2019 32383991 10.1152/ajpheart.00555.2019 PMC7311698

[CR35] Lee DH, Park JS, Lee YS, Han J, Lee DK, Kwon SW, Han DH, Lee YH, Bae SH (2020) SQSTM1/p62 activates NFE2L2/NRF2 via ULK1-mediated autophagic KEAP1 degradation and protects mouse liver from lipotoxicity. Autophagy 16(11):1949–1973. 10.1080/15548627.2020.1712108 31913745 10.1080/15548627.2020.1712108 PMC7595589

[CR36] Lee HJ, Park MJ, Heo JD, Joo BS, Joo JK (2023) Timing of hormone therapy and its association with cardiovascular risk and metabolic parameters in 4-vinylcyclohexene diepoxide-induced primary ovarian insufficiency mouse model. Gynecol Endocrinol 39(1):2247094. 10.1080/09513590.2023.2247094 37599578 10.1080/09513590.2023.2247094

[CR37] Levine B, Klionsky DJ (2004) Development by self-digestion: molecular mechanisms and biological functions of autophagy. Dev Cell 6(4):463–477. 10.1016/s1534-5807(04)00099-1 15068787 10.1016/s1534-5807(04)00099-1

[CR38] Li M, Xie L, Li Y, Liu J, Nie G, Yang H (2020) Synergistic effect of Huyang Yangkun Formula and embryonic stem cells on 4-vinylcyclohexene diepoxide induced premature ovarian insufficiency in mice. Chin Med 15:83. 10.1186/s13020-020-00362-6 32774448 10.1186/s13020-020-00362-6 PMC7405416

[CR39] Lin TY, Chan HH, Chen SH, Sarvagalla S, Chen PS, Coumar MS, Cheng SM, Chang YC, Lin CH, Leung E, Cheung CHA (2020) BIRC5/Survivin is a novel ATG12-ATG5 conjugate interactor and an autophagy-induced DNA damage suppressor in human cancer and mouse embryonic fibroblast cells. Autophagy 16(7):1296–1313. 10.1080/15548627.2019.1671643 31612776 10.1080/15548627.2019.1671643 PMC7469615

[CR40] Liu J, Wang C (2023) Lysophosphatidic acid is associated with oocyte maturation by enhancing autophagy via PI3K-AKT-mTOR signaling pathway in granulosa cells. J Ovarian Res 16(1):137. 10.1186/s13048-023-01228-9 37434211 10.1186/s13048-023-01228-9 PMC10334515

[CR41] Liu K, Ren T, Huang Y, Sun K, Bao X, Wang S, Zheng B, Guo W (2017) Apatinib promotes autophagy and apoptosis through VEGFR2/STAT3/BCL-2 signaling in osteosarcoma. Cell Death Dis 8(8):e3015. 10.1038/cddis.2017.422 28837148 10.1038/cddis.2017.422 PMC5596600

[CR42] Liu YX, Zhang Y, Li YY, Liu XM, Wang XX, Zhang CL, Hao CF, Deng SL (2019) Regulation of follicular development and differentiation by intra-ovarian factors and endocrine hormones. Front Biosci (Landmark Ed) 24(5):983–993. 10.2741/4763 30844725 10.2741/4763

[CR43] Liu W, Chen M, Liu C, Wang L, Wei H, Zhang R, Ren Z, Chen Y, Luo M, Zhao J, Jiang H, Gao F, Li W (2023) Epg5 deficiency leads to primary ovarian insufficiency due to WT1 accumulation in mouse granulosa cells. Autophagy 19(2):644–659. 10.1080/15548627.2022.2094671 35786405 10.1080/15548627.2022.2094671 PMC9851269

[CR44] Liu YX, Ke Y, Qiu P, Gao J, Deng GP (2023) LncRNA NEAT1 inhibits apoptosis and autophagy of ovarian granulosa cells through miR-654/STC2-mediated MAPK signaling pathway. Exp Cell Res 424(1):113473. 10.1016/j.yexcr.2023.113473 36634743 10.1016/j.yexcr.2023.113473

[CR45] Lum JJ, Bauer DE, Kong M, Harris MH, Li C, Lindsten T, Thompson CB (2005) Growth factor regulation of autophagy and cell survival in the absence of apoptosis. Cell 120(2):237–248. 10.1016/j.cell.2004.11.046 15680329 10.1016/j.cell.2004.11.046

[CR46] Ma Y, Zhou Y, Zhu YC, Wang SQ, Ping P, Chen XF (2018) Lipophagy Contributes to Testosterone Biosynthesis in Male Rat Leydig Cells. Endocrinology 159(2):1119–1129. 10.1210/en.2017-03020 29304246 10.1210/en.2017-03020

[CR47] Mark-Kappeler CJ, Sen N, Lukefahr A, McKee L, Sipes IG, Konhilas J, Hoyer PB (2011) Inhibition of ovarian KIT phosphorylation by the ovotoxicant 4-vinylcyclohexene diepoxide in rats. Biol Reprod 85(4):755–762. 10.1095/biolreprod.111.092742 21677306 10.1095/biolreprod.111.092742 PMC3184290

[CR48] Molero-Valenzuela A, Fontova P, Alonso-Carrillo D, Carreira-Barral I, Torres AA, Garcia-Valverde M, Benitez-Garcia C, Perez-Tomas R, Quesada R, Soto-Cerrato V. (2022). A novel late-stage autophagy inhibitor that efficiently targets lysosomes inducing potent cytotoxic and sensitizing effects in lung cancer. Cancers (Basel), 14(14). 10.3390/cancers14143387 10.3390/cancers14143387 PMC9324127 35884450

[CR49] Nishi Y, Yanase T, Mu Y, Oba K, Ichino I, Saito M, Nomura M, Mukasa C, Okabe T, Goto K, Takayanagi R, Kashimura Y, Haji M, Nawata H (2001) Establishment and characterization of a steroidogenic human granulosa-like tumor cell line, KGN, that expresses functional follicle-stimulating hormone receptor. Endocrinology 142(1):437–445. 10.1210/endo.142.1.7862 11145608 10.1210/endo.142.1.7862

[CR50] Oka SI, Byun J, Huang CY, Imai N, Ralda G, Zhai P, Xu X, Kashyap S, Warren JS, Alan MJ, Tippetts TS, Tong M, Venkatesh S, Ikeda Y, Mizushima W, Kashihara T, Sadoshima J (2021) Nampt Potentiates Antioxidant Defense in Diabetic Cardiomyopathy. Circ Res 129(1):114–130. 10.1161/CIRCRESAHA.120.317943 33928788 10.1161/CIRCRESAHA.120.317943 PMC8513534

[CR51] Orisaka M, Miyazaki Y, Shirafuji A, Tamamura C, Tsuyoshi H, Tsang BK, Yoshida Y (2021) The role of pituitary gonadotropins and intraovarian regulators in follicle development: a mini-review. Reprod Med Biol 20(2):169–175. 10.1002/rmb2.12371 33850449 10.1002/rmb2.12371 PMC8022101

[CR52] Pontifex MG, Martinsen A, Saleh RNM, Harden G, Tejera N, Muller M, Fox C, Vauzour D, Minihane AM (2021) APOE4 genotype exacerbates the impact of menopause on cognition and synaptic plasticity in APOE-TR mice. FASEB J 35(5):e21583. 10.1096/fj.202002621RR 33891334 10.1096/fj.202002621RR

[CR53] Qin J, Chen J, Xu H, Xia Y, Tang W, Wang W, Li C, Tang Y, Wang Y (2022) Low-Intensity Pulsed Ultrasound Promotes Repair of 4-Vinylcyclohexene Diepoxide-Induced Premature Ovarian Insufficiency in SD Rats. J Gerontol A Biol Sci Med Sci 77(2):221–227. 10.1093/gerona/glab242 34417809 10.1093/gerona/glab242

[CR54] Qin X, Zhao Y, Zhang T, Yin C, Qiao J, Guo W, Lu B (2022) TrkB agonist antibody ameliorates fertility deficits in aged and cyclophosphamide-induced premature ovarian failure model mice. Nat Commun 13(1):914. 10.1038/s41467-022-28611-2 35177657 10.1038/s41467-022-28611-2 PMC8854395

[CR55] Reverchon M, Rame C, Bunel A, Chen W, Froment P, Dupont J (2016) VISFATIN (NAMPT) Improves In Vitro IGF1-Induced Steroidogenesis and IGF1 Receptor Signaling Through SIRT1 in Bovine Granulosa Cells. Biol Reprod 94(3):54. 10.1095/biolreprod.115.134650 26792944 10.1095/biolreprod.115.134650

[CR56] Saiki S, Sasazawa Y, Imamichi Y, Kawajiri S, Fujimaki T, Tanida I, Kobayashi H, Sato F, Sato S, Ishikawa K, Imoto M, Hattori N (2011) Caffeine induces apoptosis by enhancement of autophagy via PI3K/Akt/mTOR/p70S6K inhibition. Autophagy 7(2):176–187. 10.4161/auto.7.2.14074 21081844 10.4161/auto.7.2.14074 PMC3039768

[CR57] Sekulovski N, Whorton AE, Shi M, Hayashi K, MacLean JAN (2020) Periovulatory insulin signaling is essential for ovulation, granulosa cell differentiation, and female fertility. FASEB J 34(2):2376–2391. 10.1096/fj.201901791R 31908002 10.1096/fj.201901791R PMC7781071

[CR58] Shao T, Ke H, Liu R, Xu L, Han S, Zhang X, Dang Y, Jiao X, Li W, Chen ZJ, Qin Y, Zhao S (2022) Autophagy regulates differentiation of ovarian granulosa cells through degradation of WT1. Autophagy 18(8):1864–1878. 10.1080/15548627.2021.2005415 35025698 10.1080/15548627.2021.2005415 PMC9450966

[CR59] Shin D, Ha J, Hong SB, Kang GH, Hwang DS, Bae H (2017) Schisandrae Fructus Reduces Symptoms of 4-Vinylcyclohexene Diepoxide-Induced Ovarian Failure in Mice. Evid Based Complement Alternat Med 2017:2564787. 10.1155/2017/2564787 28584559 10.1155/2017/2564787 PMC5443995

[CR60] Song ZH, Yu HY, Wang P, Mao GK, Liu WX, Li MN, Wang HN, Shang YL, Liu C, Xu ZL, Sun QY, Li W (2015) Germ cell-specific Atg7 knockout results in primary ovarian insufficiency in female mice. Cell Death Dis 6(1):e1589. 10.1038/cddis.2014.559 25590799 10.1038/cddis.2014.559 PMC4669757

[CR61] Springer LN, Tilly JL, Sipes IG, Hoyer PB (1996) Enhanced expression of bax in small preantral follicles during 4-vinylcyclohexene diepoxide-induced ovotoxicity in the rat. Toxicol Appl Pharmacol 139(2):402–410. 10.1006/taap.1996.0181 8806858 10.1006/taap.1996.0181

[CR62] Sugiura K, Su YQ, Li Q, Wigglesworth K, Matzuk MM, Eppig JJ (2010) Estrogen promotes the development of mouse cumulus cells in coordination with oocyte-derived GDF9 and BMP15. Mol Endocrinol 24(12):2303–2314. 10.1210/me.2010-0260 21047911 10.1210/me.2010-0260 PMC2999473

[CR63] Sun MX, Huang L, Wang R, Yu YL, Li C, Li PP, Hu XC, Hao HP, Ishag HA, Mao X (2012) Porcine reproductive and respiratory syndrome virus induces autophagy to promote virus replication. Autophagy 8(10):1434–1447. 10.4161/auto.21159 22739997 10.4161/auto.21159

[CR64] Sung JS, Kang CW, Kang S, Jang Y, Chae YC, Kim BG, Cho NH (2020) ITGB4-mediated metabolic reprogramming of cancer-associated fibroblasts. Oncogene 39(3):664–676. 10.1038/s41388-019-1014-0 31534187 10.1038/s41388-019-1014-0

[CR65] Takai Y, Canning J, Perez GI, Pru JK, Schlezinger JJ, Sherr DH, Kolesnick RN, Yuan J, Flavell RA, Korsmeyer SJ, Tilly JL (2003) Bax, caspase-2, and caspase-3 are required for ovarian follicle loss caused by 4-vinylcyclohexene diepoxide exposure of female mice in vivo. Endocrinology 144(1):69–74. 10.1210/en.2002-220814 12488331 10.1210/en.2002-220814

[CR66] Tang D, Feng X, Ling L, Zhang W, Luo Y, Wang Y, Xiong Z (2021) Experimental study for the establishment of a chemotherapy-induced ovarian insufficiency model in rats by using cyclophosphamide combined with busulfan. Regul Toxicol Pharmacol 122:104915. 10.1016/j.yrtph.2021.104915 33705838 10.1016/j.yrtph.2021.104915

[CR67] Thakre A, Gupta M, Magar SP, Bahiram KB, Sardar VM, Korde JP, Bonde SW, Hyder I (2021) Transcriptional and translational abundance of visfatin (NAMPT) in buffalo ovary during estrous cycle and its in vitro effect on steroidogenesis. Domest Anim Endocrinol 75:106583. 10.1016/j.domaniend.2020.106583 33249344 10.1016/j.domaniend.2020.106583

[CR68] Thiebaut AM, Buendia I, Ginet V, Lemarchand E, Boudjadja MB, Hommet Y, Lebouvier L, Lechevallier C, Maillasson M, Hedou E, Deglon N, Oury F, Rubio M, Montaner J, Puyal J, Vivien D, Roussel BD (2022) Thrombolysis by PLAT/tPA increases serum free IGF1 leading to a decrease of deleterious autophagy following brain ischemia. Autophagy 18(6):1297–1317. 10.1080/15548627.2021.1973339 34520334 10.1080/15548627.2021.1973339 PMC9225202

[CR69] Thongchot S, Singsuksawat E, Sumransub N, Pongpaibul A, Trakarnsanga A, Thuwajit P, Thuwajit C (2020) Periostin regulates autophagy through integrin alpha5beta1 or alpha6beta4 and an AKT-dependent pathway in colorectal cancer cell migration. J Cell Mol Med 24(21):12421–12432. 10.1111/jcmm.15756 32990415 10.1111/jcmm.15756 PMC7686974

[CR70] Tu H, Yang S, Jiang T, Wei L, Shi L, Liu C, Wang C, Huang H, Hu Y, Chen Z, Chen J, Li Z, Li J (2019) Elevated pulmonary tuberculosis biomarker miR-423-5p plays critical role in the occurrence of active TB by inhibiting autophagosome-lysosome fusion. Emerg Microbes Infect 8(1):448–460. 10.1080/22221751.2019.1590129 30898038 10.1080/22221751.2019.1590129 PMC6455132

[CR71] Wang P, Guan YF, Du H, Zhai QW, Su DF, Miao CY (2012) Induction of autophagy contributes to the neuroprotection of nicotinamide phosphoribosyltransferase in cerebral ischemia. Autophagy 8(1):77–87. 10.4161/auto.8.1.18274 22113203 10.4161/auto.8.1.18274

[CR72] Wang YY, Sun YC, Sun XF, Cheng SF, Li B, Zhang XF, De Felici M, Shen W (2017) Starvation at birth impairs germ cell cyst breakdown and increases autophagy and apoptosis in mouse oocytes. Cell Death Dis 8(2):e2613. 10.1038/cddis.2017.3 28182014 10.1038/cddis.2017.3 PMC5386484

[CR73] Wang XW, Yuan LJ, Yang Y, Zhang M, Chen WF (2020) IGF-1 inhibits MPTP/MPP(+)-induced autophagy on dopaminergic neurons through the IGF-1R/PI3K-Akt-mTOR pathway and GPER. Am J Physiol Endocrinol Metab 319(4):E734–E743. 10.1152/ajpendo.00071.2020 32865008 10.1152/ajpendo.00071.2020

[CR74] Williams CJ, Erickson GF (2012) Morphology and Physiology of the Ovary. In: Feingold KR, Anawalt B, Blackman MR et al. (Eds). Endotext [Internet]. https://www.ncbi.nlm.nih.gov/books/NBK278951/

[CR75] Wong PM, Feng Y, Wang J, Shi R, Jiang X (2015) Regulation of autophagy by coordinated action of mTORC1 and protein phosphatase 2A. Nat Commun 6:8048. 10.1038/ncomms9048 26310906 10.1038/ncomms9048 PMC4552084

[CR76] Wu Y, Ma C, Zhao H, Zhou Y, Chen Z, Wang L (2018) Alleviation of endoplasmic reticulum stress protects against cisplatin-induced ovarian damage. Reprod Biol Endocrinol 16(1):85. 10.1186/s12958-018-0404-4 30176887 10.1186/s12958-018-0404-4 PMC6122480

[CR77] Wu Q, Tian AL, Li B, Leduc M, Forveille S, Hamley P, Galloway W, Xie W, Liu P, Zhao L, Zhang S, Hui P, Madeo F, Tu Y, Kepp O, Kroemer G (2021) IGF1 receptor inhibition amplifies the effects of cancer drugs by autophagy and immune-dependent mechanisms. J Immunother Cancer 9(6). 10.1136/jitc-2021-002722 10.1136/jitc-2021-002722 PMC8204183 34127545

[CR78] Yan C, Zhao J, Qin Y, Zhao F, Ji L, Zhang J (2020) Overexpression of ATG4a promotes autophagy and proliferation, and inhibits apoptosis in lens epithelial cells via the AMPK and Akt pathways. Mol Med Rep 22(2):1295–1302. 10.3892/mmr.2020.11205 32626969 10.3892/mmr.2020.11205 PMC7339427

[CR79] Yang C, Tao H, Zhang H, Xia Y, Bai J, Ge G, Li W, Zhang W, Xiao L, Xu Y, Wang Z, Gu Y, Yang H, Liu Y, Geng D (2022) TET2 regulates osteoclastogenesis by modulating autophagy in OVX-induced bone loss. Autophagy 18(12):2817–2829. 10.1080/15548627.2022.2048432 35255774 10.1080/15548627.2022.2048432 PMC9673923

[CR80] Yi S, Zheng B, Zhu Y, Cai Y, Sun H, Zhou J (2020) Melatonin ameliorates excessive PINK1/Parkin-mediated mitophagy by enhancing SIRT1 expression in granulosa cells of PCOS. Am J Physiol Endocrinol Metab 319(1):E91–E101. 10.1152/ajpendo.00006.2020 32343612 10.1152/ajpendo.00006.2020

[CR81] Zhang Z, Chen Z, Liu R, Liang Q, Peng Z, Yin S, Tang J, Gong T, Liu Y (2020) Bcl-2 Proteins Regulate Mitophagy in Lipopolysaccharide-Induced Acute Lung Injury via PINK1/Parkin Signaling Pathway. Oxid Med Cell Longev 2020:6579696. 10.1155/2020/6579696 32148654 10.1155/2020/6579696 PMC7054785

[CR82] Zhang X, Zhang W, Wang Z, Zheng N, Yuan F, Li B, Li X, Deng L, Lin M, Chen X, Zhang M (2022) Enhanced glycolysis in granulosa cells promotes the activation of primordial follicles through mTOR signaling. Cell Death Dis 13(1):87. 10.1038/s41419-022-04541-1 35087042 10.1038/s41419-022-04541-1 PMC8795455

[CR83] Zhang Y, Han D, Yu X, Shao X, Zong C, Zhang M, Wang J, Liang J, Ge P (2022) MiRNA-190a-5p promotes primordial follicle hyperactivation by targeting PHLPP1 in premature ovarian failure. Front Genet 13:1034832. 10.3389/fgene.2022.1034832 36406123 10.3389/fgene.2022.1034832 PMC9669437

[CR84] Zhao R, Chen M, Jiang Z, Zhao F, Xi B, Zhang X, Fu H, Zhou K (2015) Platycodin-D Induced Autophagy in Non-Small Cell Lung Cancer Cells via PI3K/Akt/mTOR and MAPK Signaling Pathways. J Cancer 6(7):623–631. 10.7150/jca.11291 26078792 10.7150/jca.11291 PMC4466411

[CR85] Zhong L, Yang B, Zhang Z, Wang J, Wang X, Guo Y, Huang W, Wang Q, Cai G, Xia F, Zhou S, Ma S, Nie Y, Lei J, Li M, Liu P, Deng W, Liu Y, Han F, Wang J (2022) Targeting autophagy peptidase ATG4B with a novel natural product inhibitor Azalomycin F4a for advanced gastric cancer. Cell Death Dis 13(2):161. 10.1038/s41419-022-04608-z 35184132 10.1038/s41419-022-04608-z PMC8858318

[CR86] Zhou J, Yao W, Liu K, Wen Q, Wu W, Liu H, Li Q (2016) MicroRNA let-7g regulates mouse granulosa cell autophagy by targeting insulin-like growth factor 1 receptor. Int J Biochem Cell Biol 78:130–140. 10.1016/j.biocel.2016.07.008 27417237 10.1016/j.biocel.2016.07.008

