# Supplementary material for: The 4-vinylcyclohexene dioxide induced mouse ovarian premature failure is caused by down regulation of IGF1R and triggering excessive autophagy
Source: Stress Biol. 2025 Mar 21;5(1):21. doi: 10.1007/s44154-024-00197-3 (PMC11928347; doi:10.1007/s44154-024-00197-3)
Supplement: Supplementary file 1 — Supplementary Material 1. [file 44154_2024_197_MOESM1_ESM.docx]

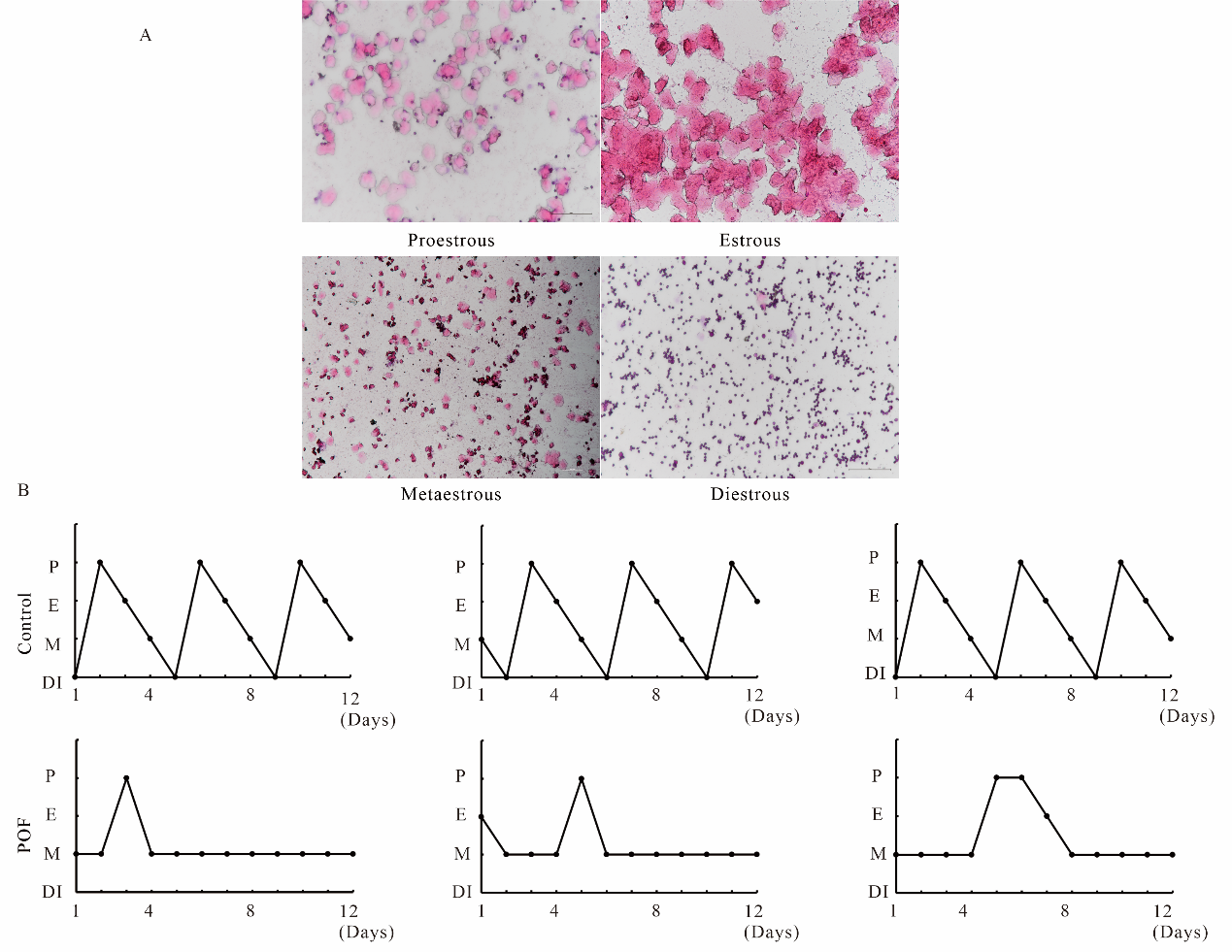
 **Figure S1**. Estrous cycle detected by vaginal smears. **(A**) Representative images of vaginal smears in different estrous cycles. Bar, 50 μm. (**B**) Estrous cycle analysis in control and POF groups. DI=Diestrous, M=Metaestrous, E=Estrous, P=Proestrous. The control group was intraperitoneally injected with the same amount of sesame oil.


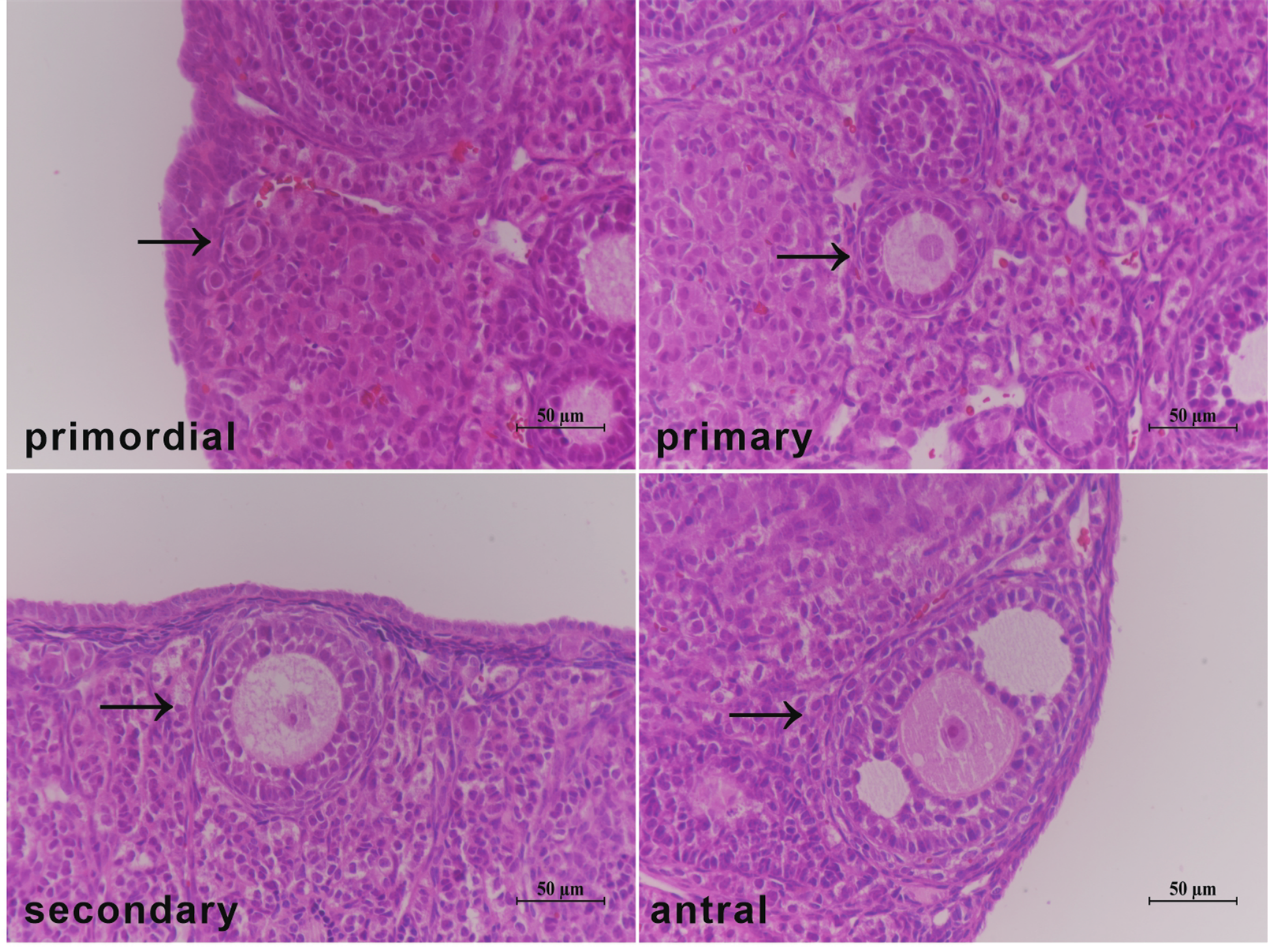


**Figure S2**. Representative images of primordial, primary, secondary, antral follicles. Bar, 50 μm.


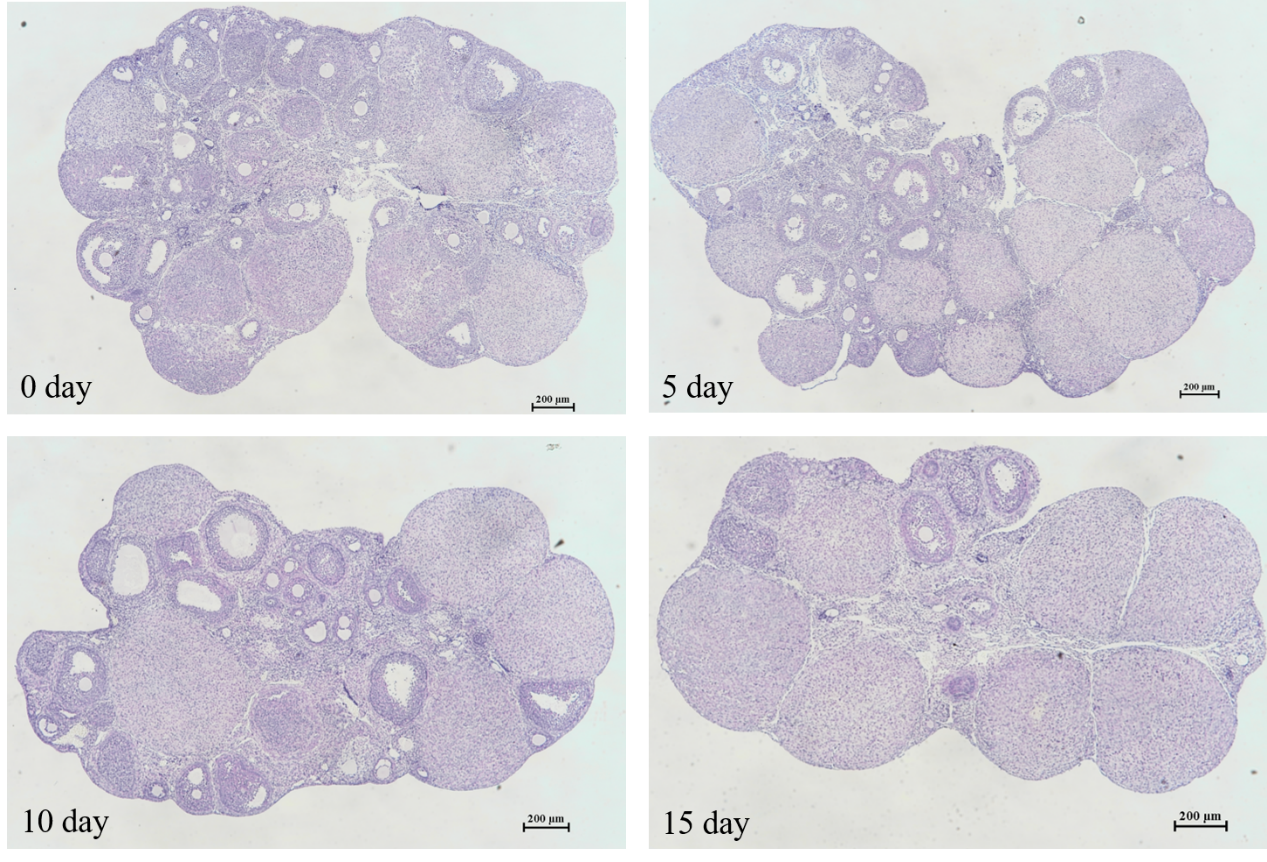


**Figure S3**. Representative images of immunohistochemical negative controls. Bar, 200 μm.


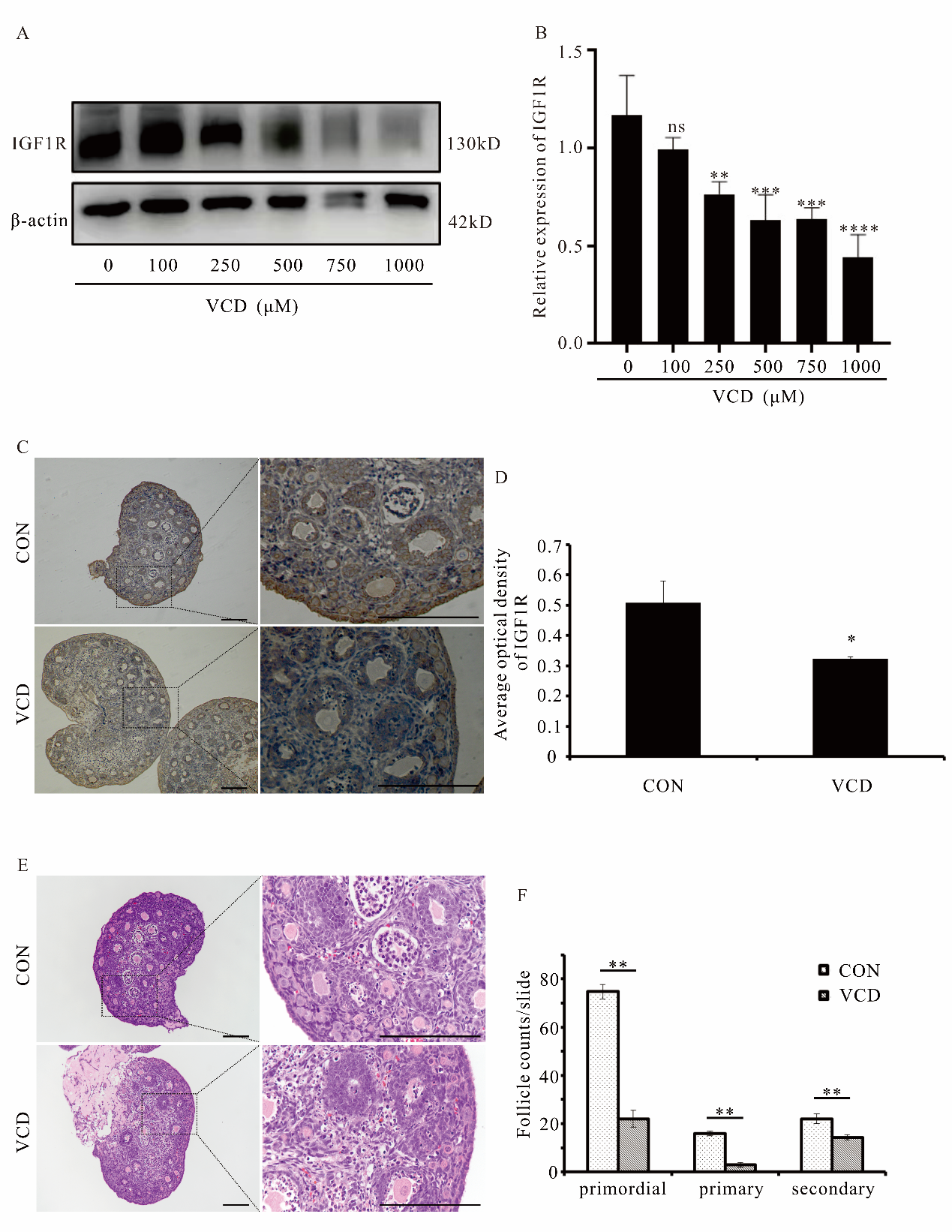
**Figure S4**. VCD down-regulated the expression of IGF1R in ovaries cultured in vitro. (**A**) Western-blotting detection of IGF1R expression of ovaries cultured in vitro treated with 0, 100, 250, 500, 750 and 1000 μM VCD for 48 h respectively. (**B**) Quantitative analysis results of Western-blotting detection of IGF1R expression in ovaries cultured in vitro(n=3/group). (**C**) Representative images of IGF1R immunohistochemistry of ovaries cultured in vitro with 0 or 250 μM VCD treatment for 48 h respectively. Bar, 200 μm. (**D**) Quantitative analysis results of average optical density (AOD) of IGF1R in immunohistochemistry(n=3/group). (**E**) Histological observation of ovaries cultured in vitro with 0 or 250 μM VCD treatment for 48 h respectively. Bar, 200 μm. (**F**) The counting results of different follicles, including primordial, primary and secondary follicles of the ovary cultured in vitro with 0 or 250 μM VCD treatment for 48 h respectively(n=5/group). Each experiment was independently repeated at least three times. The results are expressed as means ± SEM. ns *p*>0.05, **p*<0.05, ***p*<0.01, ****p*<0.001, *****p*<0.0001. The control group was treated with an equal volume of DMSO.


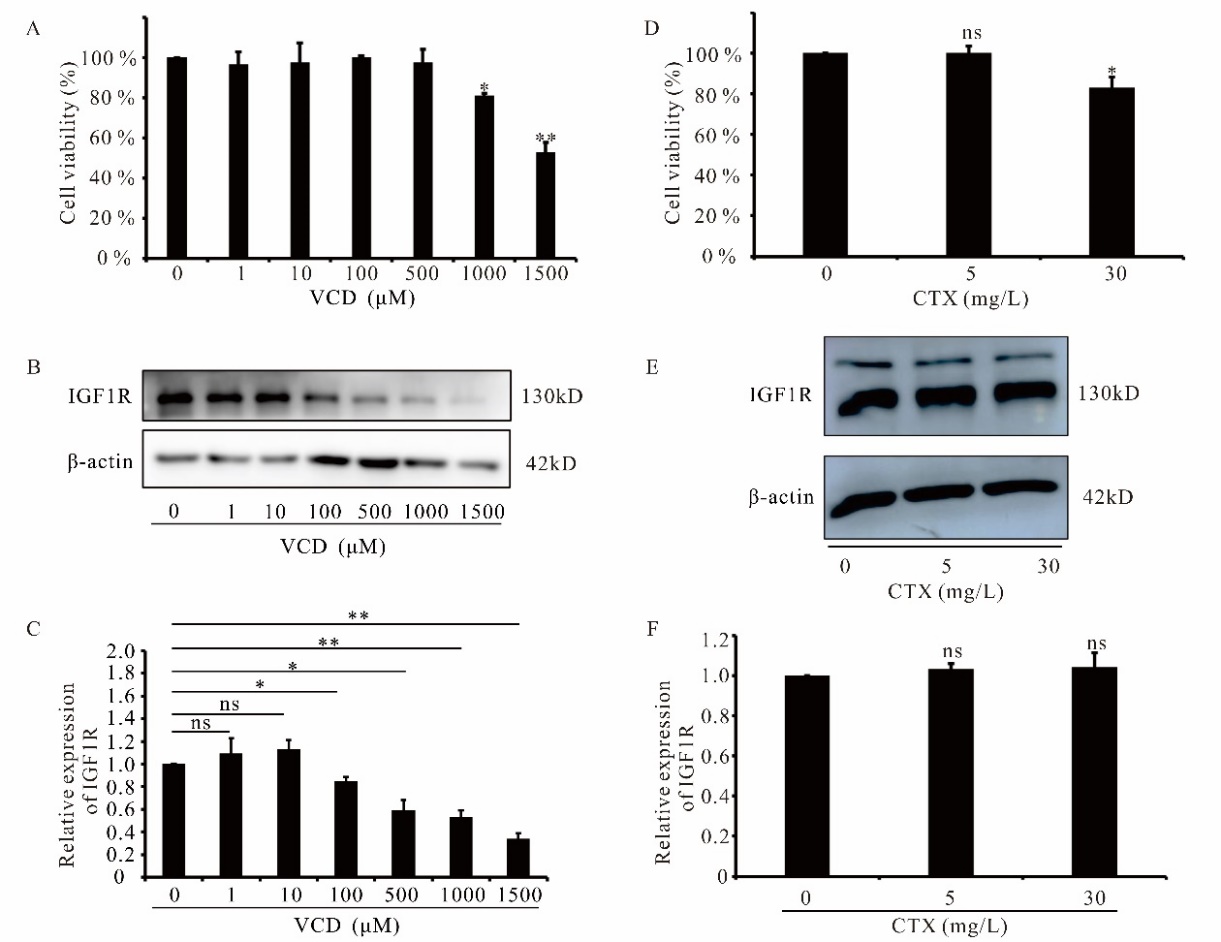


**Figure S5.** The cell viability and IGF1R expression of KGN cells treated with different concentration of VCD or CTX for 24 h. (**A**) The cell viability of KGN cells treated with different concentrations of VCD for 24 h(n=3/group). (**B**) Western-blotting detection of IGF1R expression in KGN cells treated with different concentration of VCD for 24 h. (**C**) Quantitative analysis results of Western-blotting detection of IGF1R expression in KGN cells treated with different concentrations of VCD for 24 h(n=3/group). (**D**) The cell viability of KGN cells treated with different concentration of CTX for 24 h(n=3/group). (**E**) Western-blotting detection of the expression of IGF1R in KGN cells treated with different concentrations of CTX for 24 h. (**F**) Quantitative analysis results of Western-blotting detection of IGF1R expression in KGN cells treated with different concentrations of CTX for 24 h (n=3/group). Each experiment was independently repeated at least three times. The results are expressed as means ± SEM. ns *p*>0.05, **p*<0.05, ***p*<0.01, *****p*<0.0001 vs control. The control group was treated with an equal volume of DMSO.


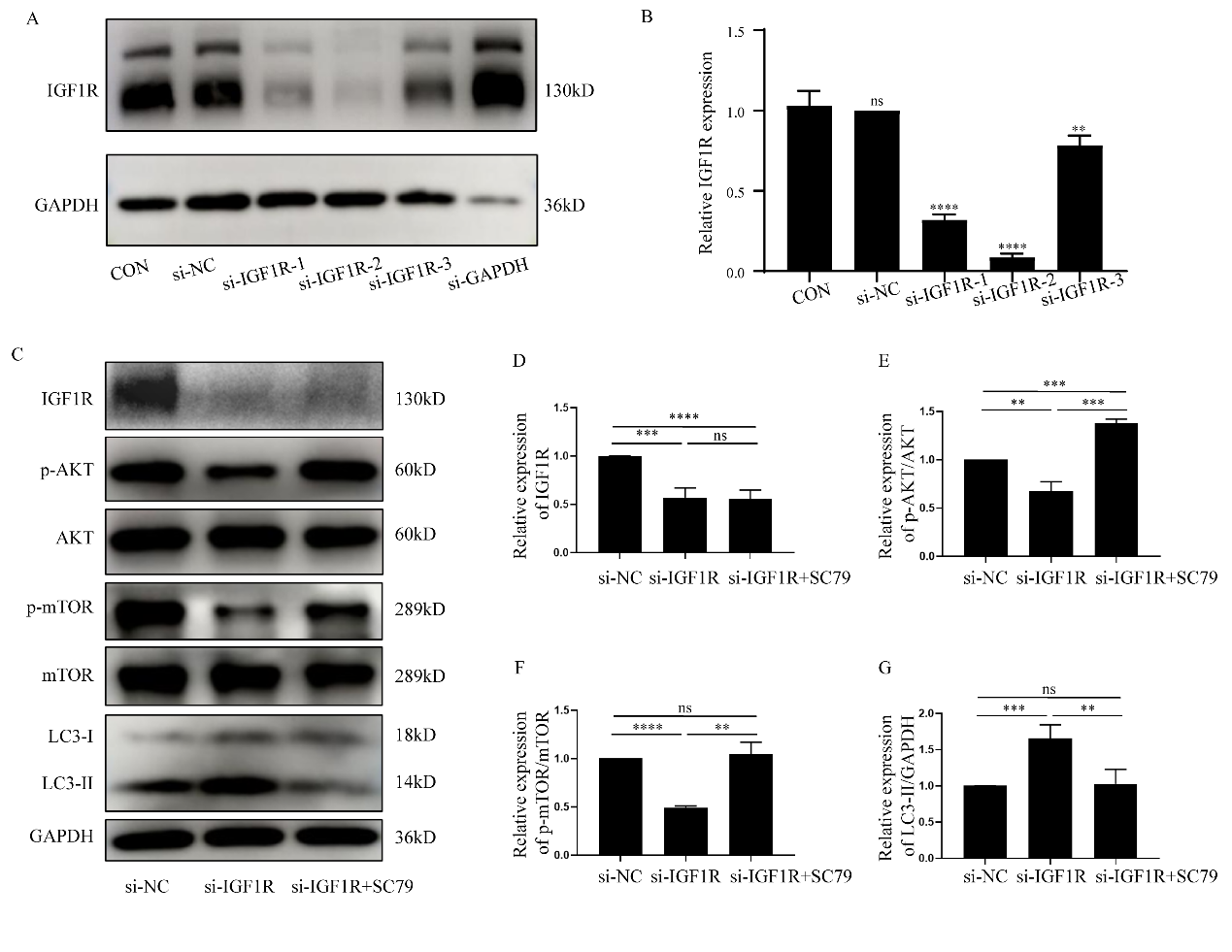
 **Figure S6**. Inhibition of IGF1R by RNAi induced autophagy in KGN cells. (**A**) Western-blotting detection of IGF1R expression in KGN cells which were transfected with 0(control), 50 nM *si-NC*, 50 nM *si-IGF1R-1*, 50 nM *si-IGF1R-2*, 50 nM *si-IGF1R-3* and 50 nM *si-GAPDH* for 48 h. (**B**) Quantitative analysis results of Western-blotting detection of IGF1R expression in KGN cells transfected with 0(control), 50 nM *si-NC*, 50 nM *si-IGF1R-1*, 50 nM *si-IGF1R-2*, 50 nM *si-IGF1R-3* and 50 nM *si-GAPDH* for 48 h(n=3/group). (**C**) Western-blotting detection of IGF1R, p-AKT/AKT, p-mTOR/mTOR and LC3-II in KGN cells treated with 50 nM *si-NC*, 50 nM *si-IGF1R* alone or with 10 μM SC79 for 24 h. (**D**) Quantitative analysis results of Western-blotting detection of IGF1R in KGN cells(n=4/group). (**E**) Quantitative analysis results of Western-blotting detection of p-AKT/AKT in KGN cells (n=3/group). (**F**) Quantitative analysis results of Western-blotting detection of p-mTOR/mTOR in KGN cells (n=3/group). (**G**) Quantitative analysis results of Western-blotting detection of LC3-II in KGN cells (n=4/group). Each experiment was independently repeated at least three times. The results are expressed as means ± SEM. ***p*<0.01, ****p*<0.001, *****p*<0.0001. The control group was treated with DMEM/F12 medium supplemented with 10% FBS.


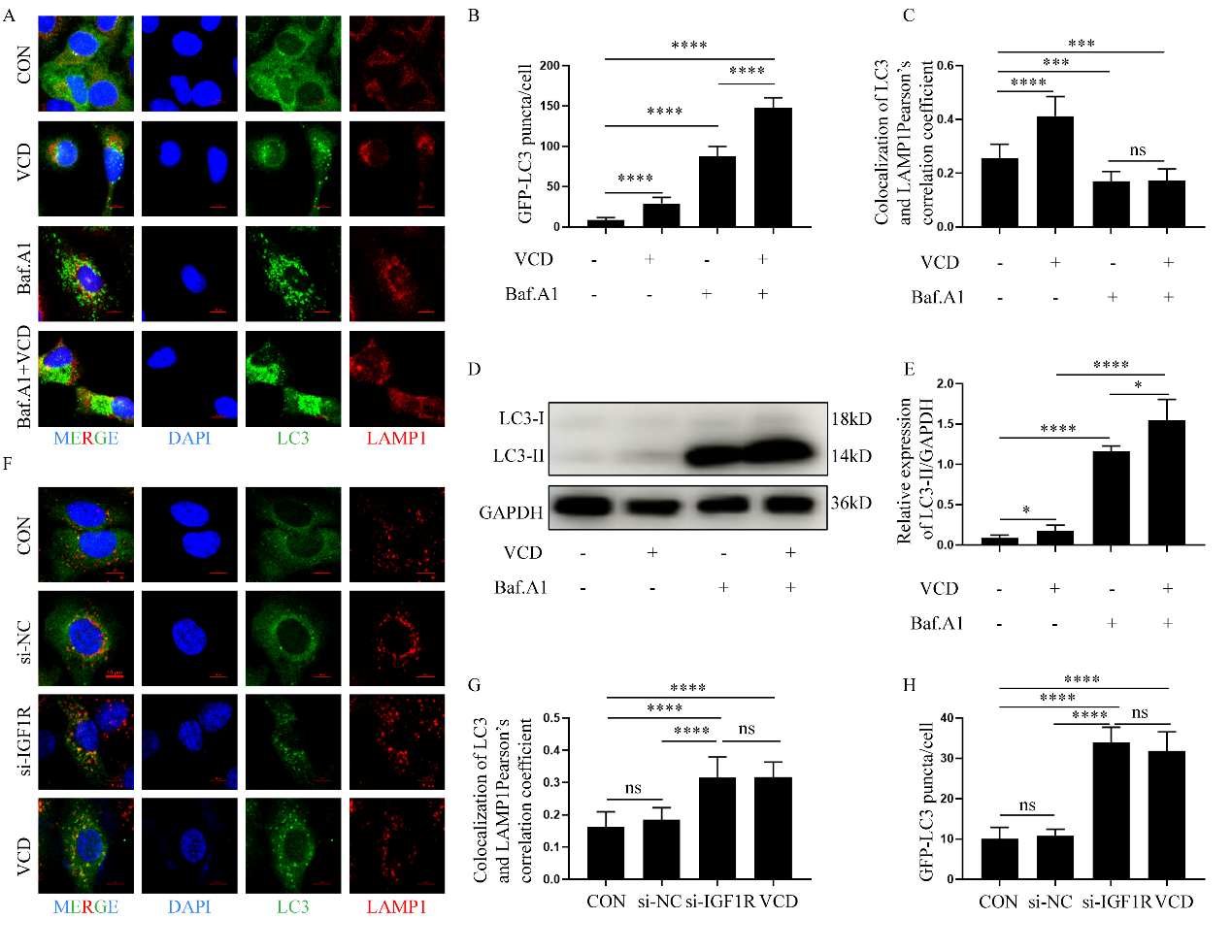


**Figure S7**. VCD activated autophagy flux in KGN cells. (**A**) Immunofluorescent analysis of autophagosomes (GFP-LC3, green), lysosomes (LAMP-1, red) and nuclei (DAPI, blue) in GFP-LC3-KGN cells pretreated with or without Baf.A1 (0.1 μM) for 1 h and then treated with VCD (500 μM) for 24h. Bar, 10 μm. (**B**) The counting results of the number of GFP-LC3 puncta per cell in GFP-LC3-KGN cells pretreated with or without Baf.A1 (0.1 μM) for 1 h and then treated with VCD (500 μM) for 24h(n=15,15,14,11/group). (**C**) Pearson correlation coefficient of the autophagosomes and lysosomes in GFP-LC3-KGN cells pretreated with or without Baf.A1 (0.1 μM) for 1 h and then treated with VCD (500 μM) for 24h(n=12/group). (**D**) Western-blotting detection of LC3-II in GFP-LC3-KGN cells pretreated with or without Baf.A1 (0.1 μM) for 1 h and then treated with VCD (500 μM) for 24h. (**E**) Quantitative analysis results of western-blotting detection of LC3-II in GFP-LC3-KGN cells pretreated with or without Baf.A1 (0.1 μM) for 1 h and then treated with VCD (500 μM) for 24h(n=5/group). (**F**) Immunofluorescent analysis of autophagosomes (GFP-LC3, green), lysosomes (LAMP-1, red) and nuclei (DAPI, blue) in KGN cells were transfected with 0(control), 50 nM *si-NC*, 50 nM *si-IGF1R*, and 500 μM VCD. Bar, 10 μm. (**G**) The counting results of the number of GFP-LC3 puncta per cell in KGN cells transfected with 0(control), 50 nM *si-NC*, 50 nM *si-IGF1R*, and 500 μM VCD(n=23,23,24,23/group). (**H**) Pearson correlation coefficient of the autophagosome and lysosome in KGN cells transfected with 0(control), 50 nM *si-NC*, 50 nM *si-IGF1R*, and 500 μM VCD(n=18,15,20,23/group). Each experiment was independently repeated at least three times. The results are expressed as means ± SEM. ns *p*>0.05, **p*<0.05, ****p*<0.001, *****p*<0.0001. The control group was treated with an equal volume of DMSO.


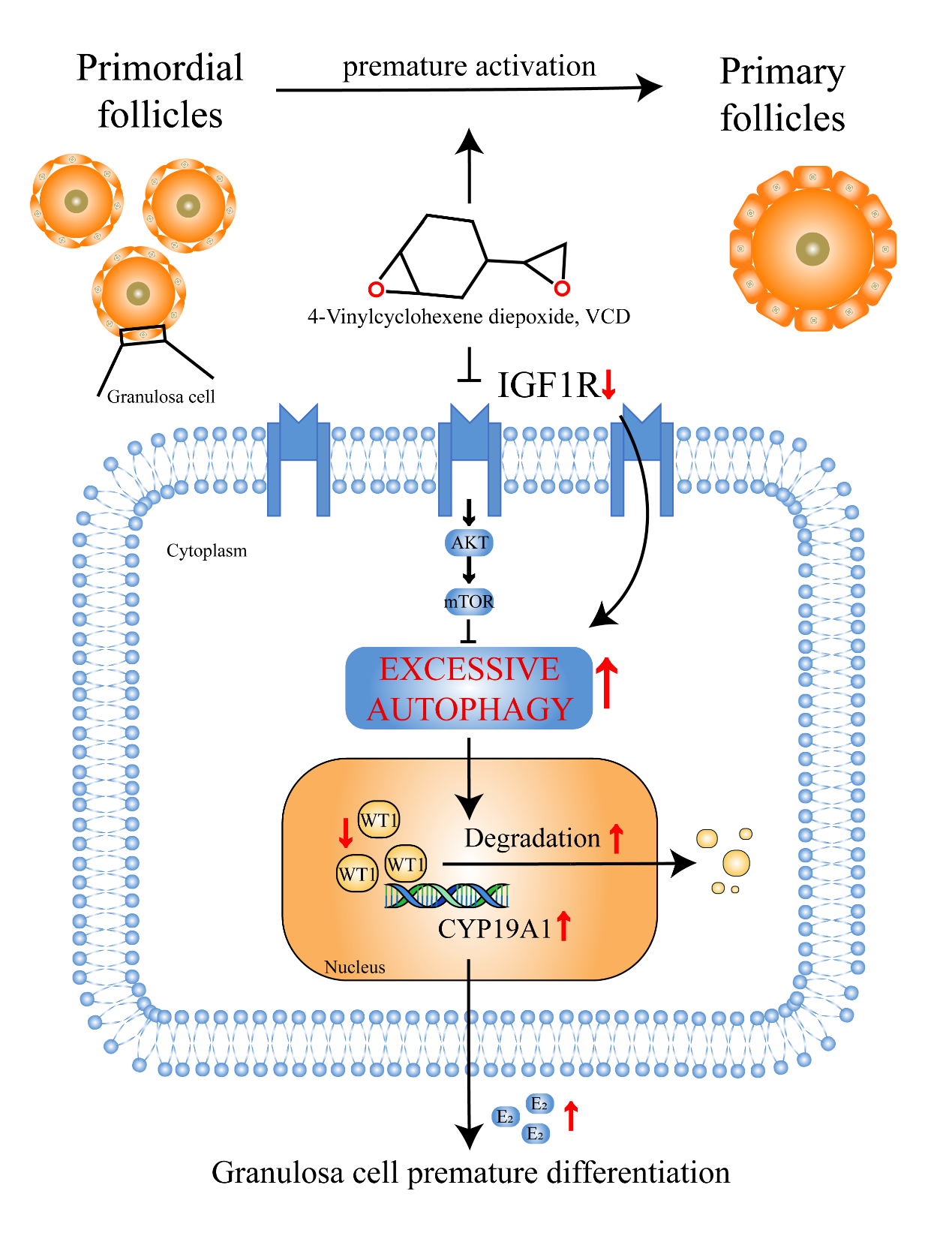
**Figure S8.** Graphical Abstract. VCD inhibited IGF1R/AKT/mTOR signaling pathway by downregulating the expression of IGF1R and triggered excessive autophagy in ovarian GCs. Further, the excessive autophagy in ovarian GCs led to the selective degradation of WT1, which promoted the expression of CYP19A1 and the secretion of E_2_, thus leading to the premature differentiation of ovarian GCs and the premature activation of primordial follicles resulting in the consumption of ovarian follicle pool.
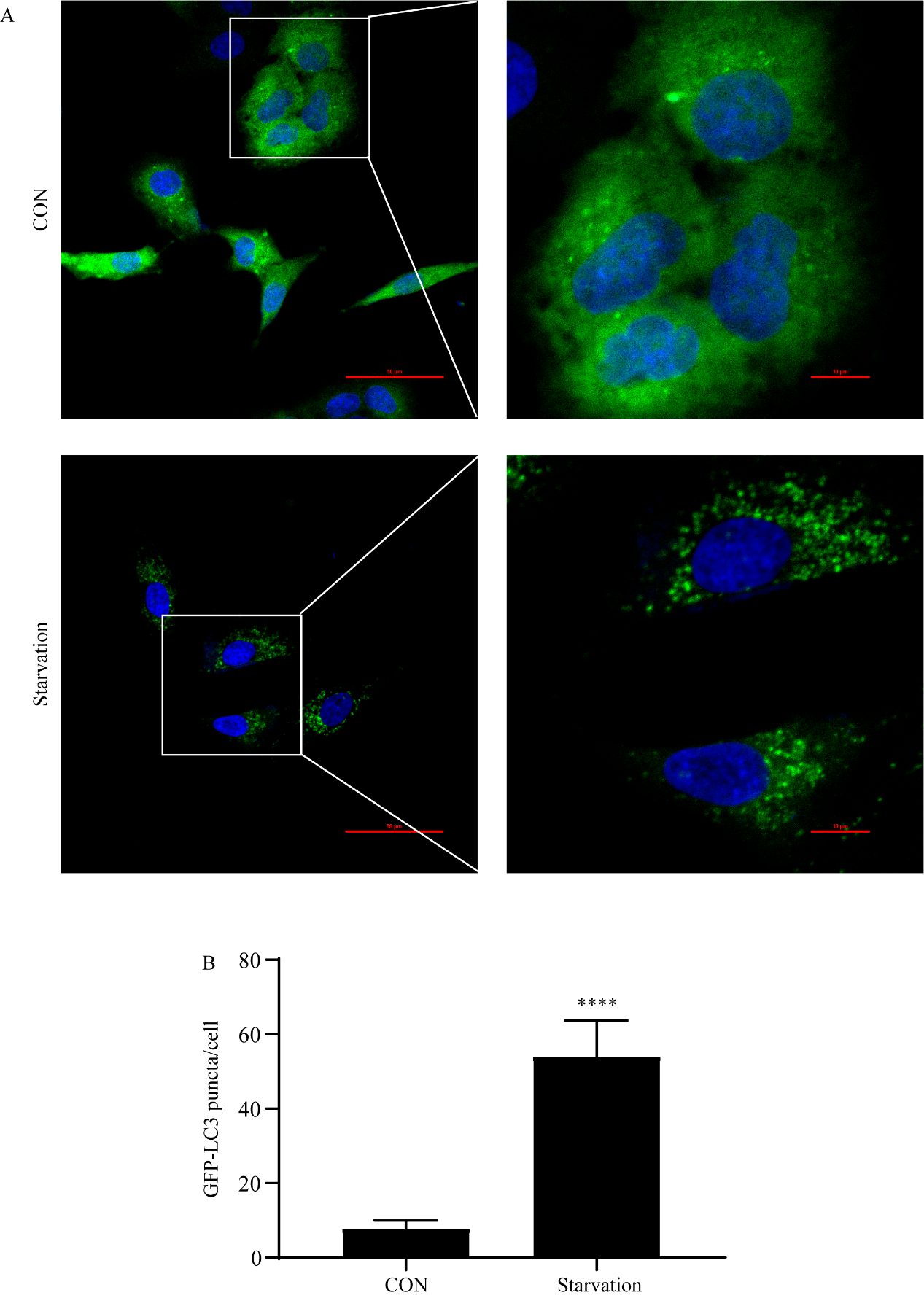
**Figure S9**. The establishment of GFP-LC3-KGN cell line. (**A**) Representative images of GFP-LC3-KGN cell after they were treated with DMEM/F12 with or without 10% FBS for 16 h. Bar, 50μm (left), 10 μm (enlarged view). (**B**) The counting results of the number of GFP-LC3 puncta per cell in KGN cells after they were treated with DMEM/F12 with or without 10% FBS for 16 h(n=14/group). Each experiment was independently repeated at least three times. The results are expressed as means ± SEM. *****p*<0.0001. The control group was treated with DMEM/F12 medium supplemented with 10% FBS.


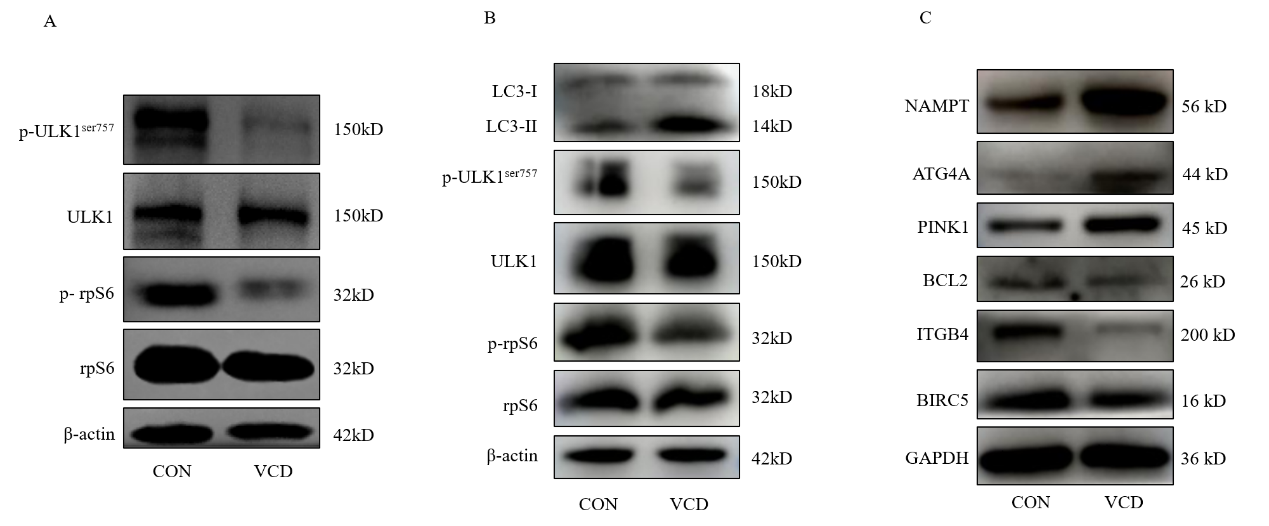


**Figure S10**. The image of Western-blotting. (**A**) VCD was intraperitoneally injected into 6-week-old female C57BL/6 mice for 15 consecutive days (160 mg/kg per day), and the ovaries were collected 45 days after VCD administration. Western-blotting detection of p-ULK1/ULK1 and p-rpS6/rpS6 expression in the ovaries. The quantitative analysis results were shown in Figure 3. (**B**) Western-blotting detection of LC3-Ⅱ，p-ULK1/ULK1 and p-rpS6/rpS6 expression in KGN cells were treated with 0 (control) or 500 μM VCD for 24 h. The quantitative analysis results were shown in Figure 4. (**C**) Western-blotting detection of NAMPT, ATG4A, PINK1, BCL2, ITGB4, BIRC5 expression in KGN cells treated with 0 (control) or 500 μM VCD for 24 h. The quantitative analysis results were shown in Figure 6.
